# VEP estimation of visual acuity: a systematic review

**DOI:** 10.1007/s10633-020-09770-3

**Published:** 2020-06-02

**Authors:** Ruth Hamilton, Michael Bach, Sven P. Heinrich, Michael B. Hoffmann, J. Vernon Odom, Daphne L. McCulloch, Dorothy A. Thompson

**Affiliations:** 1 Department of Clinical Physics and Bioengineering, Royal Hospital for Children, NHS Greater Glasgow and Clyde, Glasgow, UK; 2 College of Medical, Veterinary and Life Sciences, University of Glasgow, Glasgow, UK; 3 Eye Center, Medical Center – University of Freiburg, Faculty of Medicine, University of Freiburg, Freiburg, Germany; 4 Department of Ophthalmology, Otto-von-Guericke University, Magdeburg, Germany; 5 Center for Behavioral Brain Sciences, Magdeburg, Germany; 6 Departments of Ophthalmology and Neuroscience, School of Medicine, West Virginia University, Morgantown, WV USA; 7 School of Optometry and Vision Science, University of Waterloo, Waterloo, ON Canada; 8 The Department of Clinical and Academic Ophthalmology, Great Ormond Street Hospital for Children, London, UK; 9 University College London Great Ormond Street Institute of Child Health, London, UK

**Keywords:** Systematic review, VEP, Visual acuity, Spatial frequency limit, ISCEV, Threshold, Sweep VEP

## Abstract

**Purpose:**

Visual evoked potentials (VEPs) can be used to measure visual resolution via a spatial frequency (SF) limit as an objective estimate of visual acuity. The aim of this systematic review is to collate descriptions of the VEP SF limit in humans, healthy and disordered, and to assess how accurately and precisely VEP SF limits reflect visual acuity.

**Methods:**

The protocol methodology followed the PRISMA statement. Multiple databases were searched using “VEP” and “acuity” and associated terms, plus hand search: titles, abstracts or full text were reviewed for eligibility. Data extracted included VEP SF limits, stimulus protocols, VEP recording and analysis techniques and correspondence with behavioural acuity for normally sighted healthy adults, typically developing infants and children, healthy adults with artificially degraded vision and patients with ophthalmic or neurological conditions.

**Results:**

A total of 155 studies are included. Commonly used stimulus, recording and analysis techniques are summarised. Average healthy adult VEP SF limits vary from 15 to 40 cpd, depend on stimulus, recording and analysis techniques and are often, but not always, poorer than behavioural acuity measured either psychophysically with an identical stimulus or with a clinical acuity test. The difference between VEP SF limit and behavioural acuity is variable and strongly dependent on the VEP stimulus and choice of acuity test. VEP SF limits mature rapidly, from 1.5 to 9 cpd by the end of the first month of life to 12–20 cpd by 8–12 months, with slower improvement to 20–40 cpd by 3–5 years. VEP SF limits are much better than behavioural thresholds in the youngest, typically developing infants. This difference lessens with age and reaches equivalence between 1 and 2 years; from around 3–5 years, behavioural acuity is better than the VEP SF limit, as for adults. Healthy, artificially blurred adults had slightly better behavioural acuity than VEP SF limits across a wide range of acuities, while adults with heterogeneous ophthalmic or neurological pathologies causing reduced acuity showed a much wider and less consistent relationship. For refractive error, ocular media opacity or pathology primarily affecting the retina, VEP SF limits and behavioural acuity had a fairly consistent relationship across a wide range of acuity. This relationship was much less consistent or close for primarily macular, optic nerve or neurological conditions such as amblyopia. VEP SF limits were almost always normal in patients with non-organic visual acuity loss.

**Conclusions:**

The VEP SF limit has great utility as an objective acuity estimator, especially in pre-verbal children or patients of any age with motor or learning impairments which prevent reliable measurement of behavioural acuity. Its diagnostic power depends heavily on adequate, age-stratified, reference data, age-stratified empirical calibration with behavioural acuity, and interpretation in the light of other electrophysiological and clinical findings. Future developments could encompass faster, more objective and robust techniques such as real-time, adaptive control.

**Registration:**

International prospective register of systematic reviews PROSPERO (https://www.crd.york.ac.uk/PROSPERO/), registration number CRD42018085666.

## Introduction

Visual acuity, the threshold for resolving high contrast detail by the visual system, is an important clinical assessment, typically measured using subjective tests such as naming letters or symbols on calibrated charts or estimated using behavioural tests based on looking, pointing or matching. These tests require the patient to have adequate cognitive and motor function and to comply with the test process.

Visual evoked potentials (VEPs) are used in patients who cannot or will not reliably complete subjective or behavioural tests and in those with difficulties in perception and recognition to aid in localising defects. VEPs can be used to measure a threshold as a proxy for, or estimate of, visual acuity: such techniques for estimating acuity have been employed for over 40 years [1, 2]. VEP measurement of spatial frequency (SF) limit is objective, requires less cognitive function or cooperation than behavioural tests and does not depend on intact motor responses. However, even if identical targets or stimuli are used, a VEP measurement of SF limit and a behavioural acuity test have not assessed the same entity. Differences include:

1. At the retina, behavioural acuity tests require only a small number of normally functioning cones to resolve a grating [3], while VEPs require contributions from the fovea and peri-fovea [4].
2. A supra-threshold stimulus may be perceived but fail to evoke a measurable VEP, at least partly due to the need for sufficient neural populations to act synchronously to generate a VEP detectable by scalp electrodes.
3. Behavioural tests are self-paced with decisions based on any brief moment of optimal retinal image quality during longer viewing periods containing accommodation and fixation fluctuations [5], while the VEP will be degraded by such fluctuations because of its requirement for sustained recording.
4. Behavioural acuity tests assess the visual system as well as higher cognitive and often motor functions (target recognised, task understood, relevant motor response such as saccades, pointing or naming), while the VEP assesses cellular activity in the visual cortex and no higher processes.
5. Behavioural acuity tests use stationary targets, while VEP stimuli are dynamic, with inherent higher visibility [6].
6. Behavioural acuity is usually defined as the turning point of a psychometric function or similar measure where stimuli can still be perceived, while a VEP SF limit is often defined as zero- or near-zero extrapolated amplitude. Extrapolation may partially address issues (1) and (2).

Such differences mean that VEP SF limits and behavioural measures of acuity are not always in close agreement. However, the agreement is sufficiently consistent and close that, with suitable regard for those patient groups and disorders which are likely to produce exceptions, VEPs are a vital complementary tool for clinical assessment of acuity and may be the only measure available when behavioural testing is not possible or reliable. Systematic differences between behavioural and VEP techniques can be accounted for with appropriate conversion factors.

The aim of this systematic review is to gather and synthesise evidence to address these questions:

1. What are typical VEP SF limits in humans, in health and in disease, and how are these measured?
2. How accurately do VEP SF limits reflect visual acuity, i.e. what is the typical difference between VEP SF limits and behavioural acuity measured in the same subjects?
3. How precise are VEP SF limits, i.e. what is the typical variability?

## Methods

### Protocol and registration

This systematic review study protocol was registered with the international prospective register of systematic reviews (PROSPERO), registration number CRD42018085666. Methodology is reported according to the Preferred Reporting Items for Systematic Reviews and Meta-Analyses (PRISMA) statement [7]. Risk of bias is not assessed, as there is no standard outcome measure being compared: the greatly heterogeneous nature of the included studies preclude meaningful comparison of quality. However, factors such as number of subjects included and robustness of techniques employed are qualitatively discussed.

### Eligibility criteria, data sources and search strategies

We included articles, conference proceedings or dissertations which describe VEPs used to measure visual acuity in humans of any age, whether patients or healthy individuals. Languages were restricted to those understood by the authors, i.e. English, German and French. Exclusion criteria were: (1) meeting abstracts, review articles or editorials; (2) animal studies; (3) VEPs for communication, e.g. for brain–computer interface (4) higher-level event-related potentials; (5) VEPs used to measure thresholds other than spatial frequency, e.g. contrast sensitivity, stereoacuity, vernier or hyperacuity, colour or motion thresholds.

Two reviewers (RH, VO) independently and systematically searched MEDLINE, EMBASE, PsycINFO and ProQuest for studies published between 1975 and May 2019. MeSH terms or equivalent keywords were (“VEP” or “VECP” or “VER” or “visual evoked potential” or “visual evoked cortical potential” or “visual evoked response”) and (“acuity” or “visual acuity” or “threshold” or “spatial frequency” or “spatial threshold” or “sweep” or “swept” or “step” or “stepwise”). This search was supplemented by all authors with hand searching, e.g. reference sections of articles, reviews, book chapters, conference proceedings and monographs. Review articles or other pertinent articles pertaining to VEP SF limits were noted separately in order to capture and compare their conclusions.

### Study selection and data extraction

**Fig. 1:**
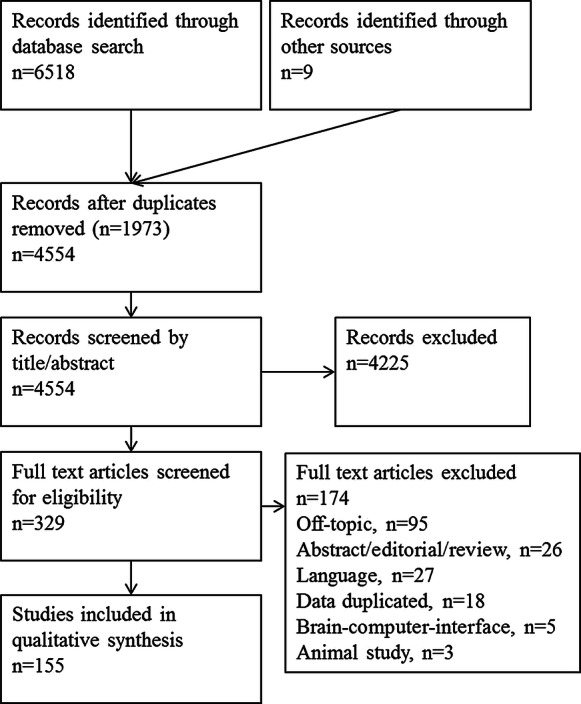
PRISMA diagram illustrating systematic review process of literature search, screen, inclusion and exclusion

Titles and abstracts were screened to identify potentially eligible studies for inclusion. Where necessary, the full text was reviewed to determine whether a study met the inclusion criteria. Data were extracted from included studies (Fig. 1) using a standardised template. Extracted information included: study design, participant demographics, details of VEP stimulation, acquisition and analysis, details of any concomitant behavioural acuity tests and main findings. Inclusion/exclusion decisions and data extraction for each study were independently reviewed by one author (RH), and any conflicting decisions were resolved through discussion.

### Synthesis of results and summary measures

Where it was possible to compare studies, typical adult VEP SF limits were noted, with summarised findings for effects of different stimulus and acquisition parameters, and threshold estimation techniques. Correspondence of typical adult VEP SF limits with behavioural thresholds (either psychophysically to stimuli identical to the VEP stimuli or using clinical acuity tests) was compiled. VEP SF limits for typically developing infants and children were used to map VEP SF limit maturation; where concomitant behavioural acuities were also measured, correspondence between VEP SF limits and behavioural thresholds was compiled.

Studies reporting the effects of poorer-than-normal acuity on the VEP SF limit were documented, including healthy adults with artificially degraded vision and adult and paediatric patients with ophthalmic or neurological pathologies. Specific note was made of evidence supporting the extent of disparity between VEP SF limit and behavioural acuity for particular conditions.

For some studies, data were available only in figures, rather than explicitly stated in tables or text: if possible, such data were extracted using web-based tools [8]. Extracted data were summarised in tabular form. If numerical pooling was not possible, we generated a set of statements to represent the body of literature reviewed.

To aid clarity, terminology conventions were observed for descriptions of acuity and related measures: “good”, “better”, “poor” or “poorer” were used in preference to “high”, higher”, “low” or “lower”, since some units, e.g. log_10_ of the minimum angle of resolution (MAR) (logMAR), are such that low numerical values are attributed to high performance acuities. Pattern element size was described as “coarse” or “fine” in preference to “high” or “low” since SF units, e.g. cycles per degree (cpd), and element size units, e.g. minutes of arc (′) have an inverse relation, and therefore opposite meanings of “low” and “high”.

For clarity, the most commonly used spatial patterns for evoking VEPs are described in Table 1, including formulae for calculating their fundamental SF (SF_f_) in cpd, and expressing VEP SF limits on a logMAR scale. A checkerboard’s SF_f_ is one check diagonal [9, 10]: if checkwidth is used instead of diagonal, the checkerboard's SF_f_ is underestimated by 0.15 log units. Where this was evident in data extracted from studies employing checkerboards, values were adjusted.

**Table 1:** Common pattern VEP stimuli: definitions, and formulae for conversion between spatial frequency units

Spatial pattern	Illustration	Fundamental spatial period (angular, minutes of arc (′))	Fundamental spatial frequency (SF_f_, cpd)	Log scale, i.e. logMAR if a threshold	Notes
Sinusoidal grating		One cycle = separation of 2 neighbouring luminance peaks or troughs = *X*	${\text{SF}}_{\text{f}} = \frac{60}{X}$	$= \log_{10} \left[ {\frac{X}{2}} \right]$	No higher SF harmonics
Square-wave grating (bars)		One cycle = a bar pair = 2 × barwidth (*w*_*b*_)	${\text{SF}}_{\text{f}} = \frac{60}{{2 \times w_{b} }}$	$= \log_{10} \left[ {w_{b} } \right]$	Multiple higher SF harmonics
Checkerboard		One cycle = a check diagonal $= \sqrt 2 \times$ checkwidth (*w*_*c*_)	${\text{SF}}_{\text{f}} = \frac{60}{{\sqrt 2 \times w_{c} }}$	$= \log_{10} \left[ {\frac{{\sqrt 2 \times w_{c} }}{2}} \right]$	Fundamental SF is obliquely oriented Multiple higher SF harmonics

Note logarithmic scale units for describing a pattern’s SF, even at VEP SF limit, does not necessarily equate to that behavioural acuity as logMAR

Multiple terms have been used to describe the performance limit as measured by VEPs, e.g. VEP SF threshold, VEP acuity, VEP acuity estimate, etc.: here, we have elected to use the term “VEP SF limit”.

## Results

### Included studies

The process of literature review including numbers of records searched, screened, included and excluded is shown in Fig. 1. A total of 329 full-text articles were screened and given a hashtag number: 155 of these which met the criteria specified in “Methods” section were included in the systematic review. These 155 are indicated in the reference list with their hashtag number at the end of the entry, e.g. (#24): corresponding reference numbers can be found via a text search function. The hashtags also appear in several figure legends and tables.

### Overview of VEP SF limit techniques

Techniques have converged somewhat over the decades. Stimuli are usually medium-to-high contrast (40–100%, Michelson), black and white patterns with moderately high (> 40 cd m^−2^) mean luminance. Both checkerboards and gratings (horizontal and vertical) are widely used. Field size shows high variability depending on the application, e.g. adult or infant studies. Most studies employ steady-state VEPs (ssVEPs—frequency components with constant amplitude and phase [11]), high stimulation rates and frequency domain analysis for fast, objective signal detection. A minority of studies describe transient VEPs subjectively analysed in the time domain. The underlying brain mechanisms return to a resting state before each re-stimulation for transient VEPs and so around 1 min of constant fixation is needed per stimulus condition for an adequately reproducible recording.

Single channel recordings are most commonly reported, with an active electrode placed over the occipital cortex, and a reference electrode often placed close by. Enhanced success with recovering small VEPs has been reported with Laplacian-type montages of three occipital electrodes closely placed. Given that analysis is conducted in the frequency domain, amplifier bandpass is usually kept relatively open. Most commonly, a discrete Fourier transform (DFT) is applied at the stimulation frequency (Hz for on/offsets; rps for reversal stimuli). The significance of the response at the stimulation frequency for each pattern size is objectively decided by adequate signal-to-noise ratio (SNR) or by a statistic based on phase, or on magnitude, or on combined magnitude and phase reaching significance.

Most commonly, these data are then used to derive a post hoc VEP SF limit by plotting a magnitude versus SF function, often on linear–linear plots. This function typically shows a descending, approximately linear limb at the finest stimuli: significant data points on this limb are fitted with a regression line which is extrapolated to some baseline, usually zero magnitude or a noise level, to estimate the VEP SF limit. An alternative technique, sometimes employed when linear extrapolation is not feasible, is to declare the VEP SF limit as the finest SF evoking a significant VEP.

While earlier workers sampled VEPs to continuously changing SF, a true “sweep VEP”, current implementations are based on discrete SF steps, with each SF presented for around a second. The term “sweep VEP” is nonetheless widely retained, although “stepwise sweep” or “sampled sweep” is also used synonymously. SFs are sampled either linearly or exponentially (linearly spaced on a logarithmic scale). The number of SFs presented varies widely, from as few as three or four to about 20. Their temporal order is almost always fixed, with the direction of change sometimes from fine-to-coarse, but more often from coarse-to-fine patterns. Occasionally a quasi- or pseudo-randomised order is used but real-time, adaptive techniques are seldom implemented.

### VEP SF limits in normally sighted adults

**Fig. 2:**
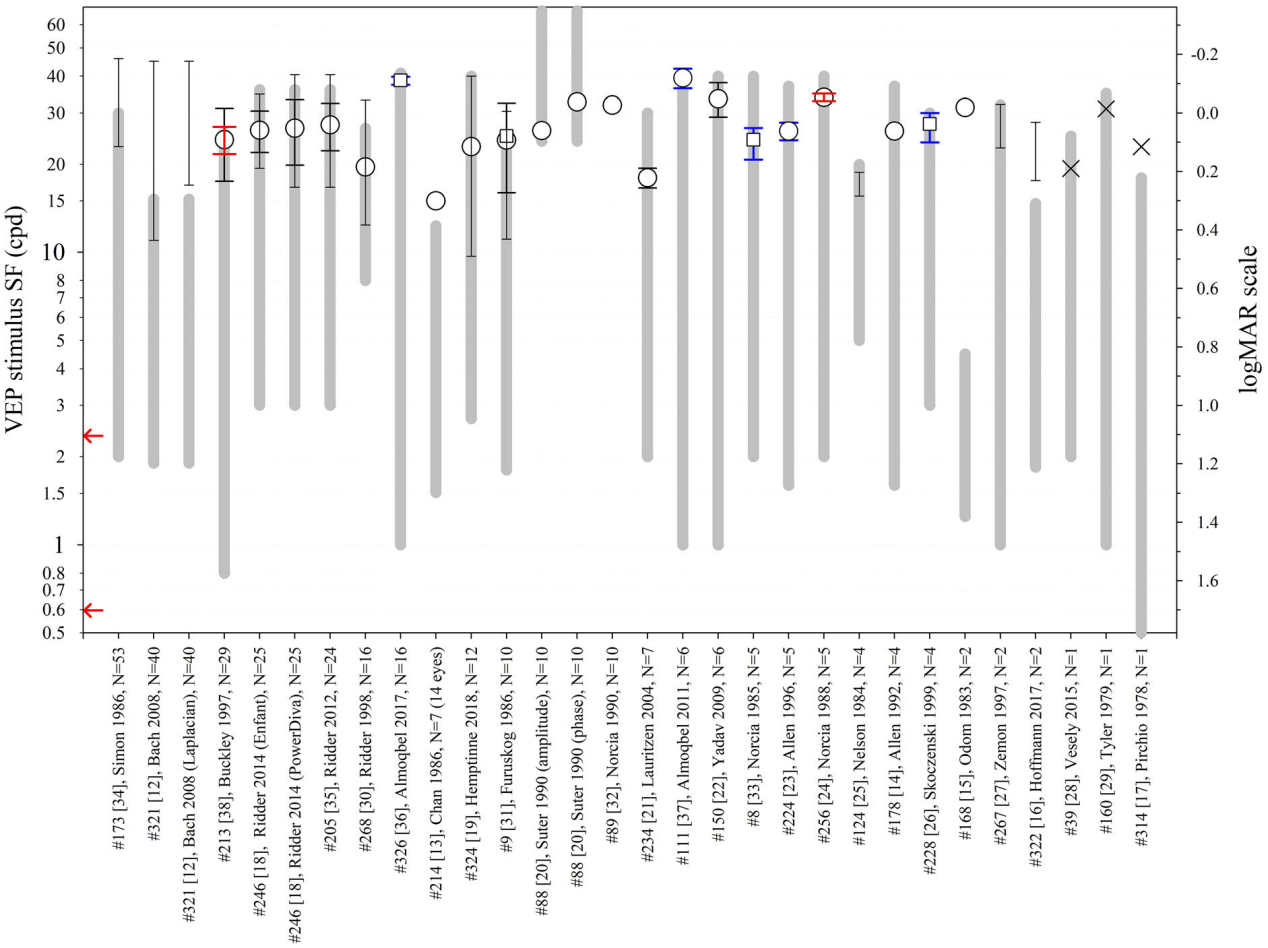
VEP stimuli SF ranges and limits (cpd) from healthy adults with normal or corrected-to-normal visual acuity as reported in 27 studies. The x-axis lists the publication and the number of subjects. Circles: mean. Squares: geometric mean. × : individual values. Lines with narrow ends: range. Black error bars (wide ends): SD. Red error bars: 95% confidence interval of mean. Blue error bars: SEM. Thick grey lines indicate the range of spatial frequencies presented or available. Arrows at the SF axis indicate the two ISCEV standard checkwidths, 60′ and 15′ (0.71 and 2.8 cpd; 1.63 and 1.026 in logMAR units) [66]

To establish typical VEP SF limits, included papers were reviewed for those which reported VEP SF limits in normal or healthy adults wearing any required refractive correction. Papers were included where VEP SF limits were stated or could be extracted. Average limits vary from 15 to 40 cpd. Methods are too diverse to relate different stimulation or analysis techniques to differences in limits (Fig. 2). Where extrapolation techniques are used, VEP SF limits can be beyond the finest SF viewed [12–17]. Many of the studies may include a ceiling effect since the VEP SF limit was beyond the range of tested SFs. Where SFs viewed or available extend to finer values than the VEP SF limit, i.e. bracket the electrophysiological limit and presumably eliminate the possibility of a ceiling effect, average VEP SF limits are slightly higher [18–33]. Several studies give examples of individuals with VEP SF limits of ≥ 40 cpd [12, 18, 19, 34–37] suggesting this as a suitable upper limit for subjects where a normal VEP SF limit is possible.

We identified only one study which described VEP SF limits in older adults [38]. Both behavioural acuity and VEP SF limits peaked around 20 years of age (Landolt C − 0.20 logMAR, VEP SF limit 44 cpd (− 0.17 logMAR)), before gradually declining at similar rates: the oldest age group was around 70 years of age and had average Landolt C acuity of 0.11 logMAR and average VEP SF limit of 22 cpd (0.14 logMAR) [38].

#### Effect of stimulus variables on VEP SF limits in normally sighted adults

- Temporal frequency

VEP amplitude is temporally as well as spatially tuned: for fine checkerboards, amplitudes are largest at slow reversal rates (≈ 7 Hz or 14 rps) but for coarse checkerboards, amplitudes are enhanced at faster reversal rates (7–11 Hz or 14–22 rps). A similar effect is seen for sinusoidal gratings, tuned at around 5–9 Hz (10–18 rps) and at around 14–22 Hz (27–44 rps) for fine and coarse gratings, respectively [39]. Despite this, there is broad agreement that VEP SF limits are relatively unaffected by stimulation rates. Similar VEP SF limits were found for 12 rps and 15 rps using reversing sinusoidal gratings and similar contrast and field size, albeit differing mean luminances (40 [20] vs. 50 cd m^−2^ [37]). No difference in extrapolated VEP SF limits were found for reversal rates from 2 to 40 rps (sinusoidal grating, 80 cd m^−2^, circular 4°) [23]. Similarly, VEP SF limits for 10 versus 2 rps differed by less than 2.5′ (checkerboard and sinusoidal gratings; 15 and 50% contrast, 50 cd m^−2^ , 8 × 11°) [40]. Comparing very different reversal rates, 3 versus 43 rps, did find a difference with VEP SF limits of 17 versus 10 cpd using a true sweep of sinusoidal gratings (75% contrast, 50 cd m^−2^, 8.25 × 11.5°) [25]. In terms of success rates (sufficient data for extrapolation), an intermediate reversal rate of 15 rps was slightly better than 12 or 20 rps (reversing sinusoidal gratings, 90% contrast, 50 cd m^−2^ luminance, 6 × 6°, 4 × 4° and 2 × 2° fields) [37]. VEP amplitude at a single checksize (5.5′) was more responsive with acuity, i.e. changed more per acuity-unit-change, for slower (3 or 6 rps) than for faster (12 rps) reversal rates over a small range of near-normal acuities (80% contrast, 31 cd m^−2^ luminance, 8.4 × 6.5° field) [41]. Finally, in 24 normally sighted adults and 35 amblyopic subjects, VEP SF limits closely matched for on/offset grating stimuli at 3.75 and at 15 Hz (80% contrast, 110 cd m^−2^, 12 × 9°), and there were no significant differences in regressions of the VEP SF limits with corresponding psychophysical grating acuities at the two different temporal frequencies (3.75 Hz on/off vs. 15 Hz on/off) [42].

b. Mean spatial luminance

Generally, higher luminance stimuli give better VEP SF limits. Increasing luminance from 0.01 through 10 to 100 cd m^−2^ improved VEP SF limits from ~ 3 cpd to ~ 18 cpd to ~ 26 cpd (sinusoidal gratings, 12 rps, 80% contrast) [14]. Increasing mean luminance from 46 to 360 cd m^−2^ improved one subject’s VEP SF limit from 11 to 31 cpd; however, the luminance specificity of this effect is not clear since it was accompanied by four other stimulus changes (reducing field size from 20 × 15° to a 2° diameter disc, increasing the SF available from 12.5 to 35 cpd, increasing contrast from 80 to 90% and changing from sinusoidal to bar gratings) to bring the stimulus closer to the clinical measurement conditions [29]. Smaller changes in luminance at higher mean luminances (25, 50 and Checkerboards and sinusoidal gratings

100 cd m^−2^) did not change VEP SF limits (sinusoidal gratings, 15 rps, 90% contrast, 6.3 × 6° field) [22].

c. Contrast

Higher contrast generally results in better VEP SF limits. Increasing contrast from 20 to 100% improved VEP SF limits from ~ 11 to > 20 cpd (checkerboard, 14 rps, 11 cd m^−2^, 4.5° diameter field) [43]. Similarly, the low amplitude VEPs found in around 10% of healthy subjects could be induced to match amplitudes of the rest of the group by increasing contrast from 40 to 80% (16 Hz on/offset sinusoidal gratings, 17 cd m^−2^, 5° diameter field [44]. VEP SF limits systematically improved by up to 4.3′ for 50% versus 15% contrast (checkerboard and sinusoidal gratings; two and 10 rps, 50 cd m^−2^, 8 × 11° field size) [40].

Higher contrast does have the disadvantage of causing one or more notches—reduced amplitude VEPs at intermediate SFs—in the SF tuning curve which could cause a marked underestimate of VEP SF limit [45–47]. This notch was evident for contrasts greater than ~ 40% (reversing sinusoidal gratings, 16–48 rps, 40 cd m^−2^, 10 × 12° field), although the authors noted that high contrast (> 50%) increased SNR [48] and other workers noted a notch even at contrasts lower than 40% [49]. Lower contrast has another potential benefit as a narrower luminance range is sufficient, which is less sensitive to inaccuracies of gamma correction and thus helps avoid luminance artefacts [12]. Healthy adult viewers at least tend to find lower contrast stimuli more comfortable to watch [19].

d. Field size

As a rule of thumb, finer patterns (< 15′ element width) evoke mainly foveal VEPs, whereas coarser patterns (> 30′ element width) evoke VEPs also via extrafoveal stimulation [39]. VEP amplitudes increase with increasing field size (2–9° diameter), and more so for coarser patterns; for smaller patterns (≤ 5′), field sizes > 4° diameter do not cause increased VEP amplitude (reversing checkerboard, 14 rps, 310 cd m^−2^, 95% contrast [50]. Circular field sizes of 15–4° produced similar VEP SF limits of around 11 cpd despite generally reducing VEP amplitudes, although amplitudes to a 2° field were too low for extrapolation (reversing gratings, 24 rps, 80% contrast, 46 cd m^−2^ luminance) [29]. Similarly, field sizes of 6 × 6°, 4 × 4° and 2 × 2° did not produce different VEP SF limits (reversing sinusoidal gratings, 12 and 15 rps, 90% contrast, 50 cd m^−2^ luminance) [37]. Similarly, although VEP magnitudes were generally larger for one VEP system with a large (13 × 10°) field than for another using a smaller field (3 × 6°), the VEP SF limits did not differ. Both systems employed sinusoidal gratings of 100 cd m^−2^ and 80% contrast reversing at 15 rps, and only slightly different sets of SFs [18].

e. Pattern

Checkerboards and sinusoidal gratings are most commonly used, with square-wave gratings (bars) also described. Surprisingly, no studies have compared VEP SF limits from such stimuli. Sinusoidal gratings are simpler stimuli in that they contain a single SF and obviate the need to interpret pattern element size [40]. The sharp edges of square-wave gratings or checkerboards may provide a better accommodative stimulus [27]. No systematic VEP SF limit differences were found between horizontally and vertically oriented gratings [25, 48] although an oblique effect is evident, with poorer VEP SF limits for oblique than for orthogonal orientations [25]. Since a checkerboard’s SF_f_ is oriented obliquely [9, 10], this finding may have relevance for checkerboard VEP SF limits.

f. Reversal versus on/offset modulation

Pattern reversal, or counterphase modulation, maintains constant mean luminance and only contrast alters. Two reversals comprise one cycle, thus 16 rps = 8 Hz. The neural response to each reversal is the same, and therefore, the electroencephalogram (EEG) spectrum has only even harmonics of the stimulus frequency (½ rps) [51]. For clarity, the frequency of a pattern-reversal stimulus should be specified in rps to avoid any confusion over whether a frequency in Hz incorrectly refers to reversal rate or correctly refers to the stimulus frequency, i.e. two reversals. Pattern on/offset modulation periodically exchanges the pattern with an isoluminant grey field: differing neural responses to the onset and offset of the pattern and/or differing onset and offset durations create both even and odd harmonics in the EEG spectrum. Luminance artefacts are possible if the grey field is not matched in average spatial luminance to the patterned field.

A more marked notch at intermediate SFs was found for reversing stimuli than for on/offset stimuli [44, 52]. Strasburger and co-workers also noted that on–off modulation reflects stimulus visibility at high SF more accurately than reversal [44]. Brief onsets (e.g. 40 ms) cause the on- and off-responses to overlap, producing a larger and therefore more detectable VEP than longer onsets (e.g. 300 ms) [53, 54]. One study compared VEP SF limits to 15 Hz on/offset and 15 rps reversing stimuli in 22 normally sighted adults and 31 adults with amblyopia (80% contrast, 110 cd m^−2^, 12 × 9°). Their VEP SF limits were highly correlated (*r* = 0.79), and there were no significant differences in regressions of the VEP SF limits with corresponding psychophysical grating acuities at the two different pattern presentations [42].

g. Spatial frequency (SF) properties

SF properties include sampling range, sampling density, direction of SF change and adaptation effects. The validity of any extrapolation technique depends on adequately dense and extensive sampling of the VEP amplitude versus SF function, especially with reversing stimuli which may produce multiple peaks, i.e. a notched function. In healthy individuals at least, patterns up to 40 cpd may be required: an 8 cpd upper limit was not sufficiently high to avoid underestimation errors [33]. Even with an upper limit of 27 cpd, a ceiling effect was noted: it has been suggested that the SF range should bracket a subject’s VEP SF limit [30, 55].

**Fig. 3:**
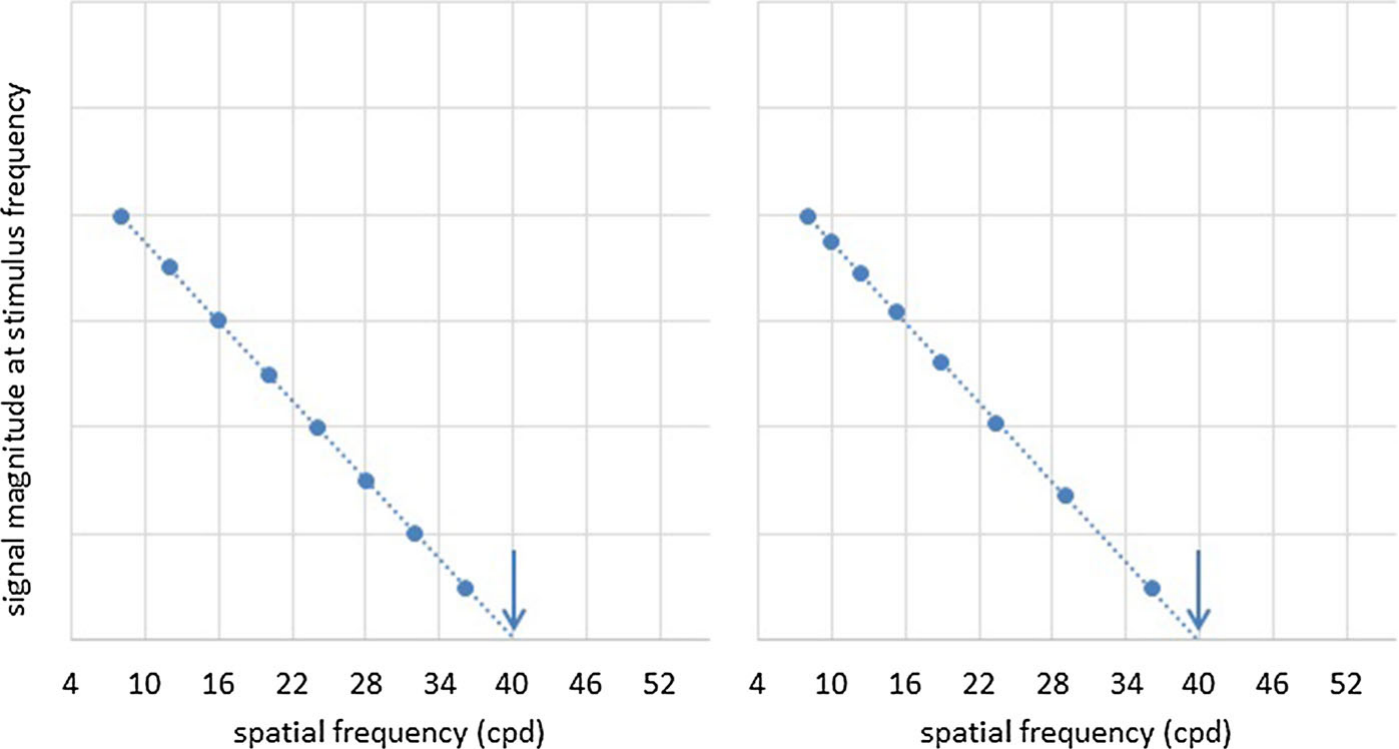
Effect of sampling—illustration of VEP SF limits for artificial data sampled linearly (left) and exponentially (right) with SF. Data show a linear relationship between VEP magnitude and SF close to threshold using linear axis scales. Linear SF sampling (left) improves sampling density close to typical adult VEP SF limit, while exponential SF sampling (right) maps more closely to psychophysical SF channels

Linear sampling^1^ of SF (equal spacing of stimuli SFs (cpd) on a linear scale) results in desirably fine sampling towards the VEP SF limit of normal adults, thus accurately representing the final slope. However, discrete steps in pixel size result in nonlinear changes in element area at the finest SFs, e.g. 1 × 1 to 2 × 2 to 3 × 3 pixels. Exponential sampling (equal spacing of stimuli SFs (cpd) on a logarithmic scale) gives equal weight to each octave of SF (Fig. 3), and results in a VEP amplitude versus SF function which corresponds with psychophysical tuning functions [56, 57]. However, spatial resolution reduces towards the acuity limit and some authors therefore describe exponential SF sampling as unsuitable for acuity measurement [29, 33]. To our knowledge, no direct comparison of exponential versus linear SF sampling has been undertaken.

The presence of SF ‘channels’ (neural populations selectively sensitive to limited but overlapping SFs taking hundreds of milliseconds to reach steady state [56, 58–60]) theoretically advocates for sequential SF presentation. Random SF sampling could stimulate a different spatial ‘channel’ with each change in SF, increasing the number of times steady state must be reached and potentially lengthening test time [33]. VEP amplitude stabilises several seconds after stimulus onset [61, 62]); however, no effect on VEP SF limit was found for stimulus durations from 1 to 8 s per stimulus [30], nor for stepwise sweeps varying in duration from 11 to 20 s [37].

The direction of SF change (coarse-to-fine or fine-to-coarse) does not appear to incur significant VEP hysteresis, i.e. minimal adaptation effect [63], perhaps due to the relatively narrow bandwidth of SF channels (≈ 1–1.4 octaves) [64] or the multiple SFs present in bar gratings and checkerboards. Studies comparing VEP SF limits obtained using coarse-to-fine and fine-to-coarse SF changes found no differences [19, 37, 65] although subjects were noted to be more attentive to coarse-to-fine stepwise sweeps [65].

In summary, suitable stimuli for VEP SF limit measurements in healthy adults have stimulation rates in the range of ≈10–24 rps for reversing stimuli or 5–12 Hz for on/offset stimuli. A large range of mean luminances have been successfully used, including values in the range of around 25–100 cd m^−2^. Contrast choice should balance the requirement for better SNR with any need to avoid a notch (reversing patterns), to avoid a luminance artefact (on/offset patterns), and to ensure viewing comfort. Field sizes > 2° are suitable. There are few data to guide the choice between sinusoidal gratings, square-wave gratings or checkerboards; grating orientation (horizontal/vertical) does not appear to affect VEP SF limits, but as with subjective thresholds, there is a small oblique effect. On/offset modulation produces VEPs which are consistently larger across all SFs than reversal modulation, but greater care is required to avoid luminance artefacts. Brief onset durations offer further amplitude enhancement over longer onset duration. The range of SFs should approach or bracket subjects’ limits for acceptable accuracy and should include sufficient data points for acceptable precision of VEP SF limit. Linear SF sampling benefits accuracy due to higher sampling close to threshold, while exponential SF sampling more closely emulates psychophysical tuning functions. Neither stimulus duration per SF condition (1–8 s), sweep duration (11–20 s) nor direction of SF change (coarse-to-fine or fine-to-coarse) appear to alter significantly VEP SF limits.

#### Effect of acquisition and analysis variables on normally sighted adult VEP SF limits

- Monocular versus binocular viewing

Specific clinical requirements determine whether VEP SF limits should be recorded monocularly or binocularly; studies of healthy adults have used either monocular or binocular viewing in approximately equal number. Limited data comparing the two viewing conditions suggest binocular VEP amplitude versus SF functions have amplitudes larger than those of monocular functions by a factor of around 1–2, but VEP SF limits are similar [48].

b. Electrode position and montage

ISCEV standard VEPs [66] require an active electrode at the midline over the occiput (Oz) referred to a distant, frontal electrode (Fz). Alternative lateral active electrode sites (PO7, O1, O2, PO8) produced similar VEP SF limits to Oz, but tended to have lower SNR and thus fewer subjects had viable threshold extrapolations [22, 37]. Averaging viable limits from five occipital channels resulted in the lowest coefficient of variation compared with using selected channels [21]. A 64-channel study using the average of all channels as the reference found Oz to be the optimal active electrode site, but noted the most sensitive zone stretches down towards the inion (Iz) and laterally to PO7, O1, O2 and PO8. Coarse patterns were often detected over right occipital/parietal positions, while fine patterns were optimally detected at midline positions [19].

VEPs are not necessarily symmetric about the midline in many individuals. Selecting data from one of two occipital channels with closely spaced active and reference electrodes (Oz–O1 and Oz–O2) based on highest SNR at peak VEP magnitude might optimise detection of an individual’s VEP SF limit [23, 24, 26, 32, 65]. This principle is extended via Laplacian electrode montages which localise evoked potential sources and improve VEP SNR using closely placed active and reference electrodes, as each carries highly coherent noise [67]. This produces good cancellation of remote noise such as eye movements or spatially diffuse noise such as EEG alpha activity. The active site, usually close to Oz for VEPs, uses the arithmetic mean of voltages from the surrounding electrodes as its effective reference voltage. For example, a Laplacian channel using a montage of Oz (active) and lateral electrodes at O1 and at O2 would use Oz voltage at the positive/active input and (½(O1 + O2)) voltage at the negative/reference input. Optimal lateral electrode positions are at 15% of half-head circumference (4–4.5 cm for normal adults) [68]. This one-dimensional Laplacian montage was faster than an Oz–Fz montage at detecting VEPs to fine patterns, e.g. those close to VEP SF limit [68]. The same Laplacian montage found similar VEP SF limits as an Oz–Fz montage, but with lower variability [12, 69]. A two-dimensional Laplacian montage (four electrodes placed orthogonally 3 cm around an active electrode site 2 cm above Iz) also enhanced SNR and improved intra-subject reliability, while eliciting comparable VEP SF limits to a traditional montage (2 cm above Iz referred to Fz) [70]. Thus, the key benefit of a Laplacian montage is enhanced SNR, especially close to threshold.

c. Criteria for VEP detection

Steady-state VEP analysis in the frequency domain, sometimes following time domain averaging, typically uses only the first harmonic (at the stimulus frequency) for pattern on/offset stimuli or second harmonic (at the reversal rate) for pattern-reversal stimuli. Including one [26, 71–77] or even more [78, 79] higher harmonics has been explored. Only one study combined harmonics for analysis, using a simple sum of the first and second harmonic magnitudes [76]. In other steady-state VEP applications, the square root of summed harmonic powers has been used to combine harmonics for a “global SNR” [80]. While this assumes that harmonics reflect a common response [81], which may not necessarily be the case [82], using a global SNR to improve diagnostic utility of VEP SF limits rather than to probe pathophysiological processes may be justified.

Signal detection commonly includes a criterion of SNR ≥ 3 [14, 18, 21–23, 26, 28, 32, 33, 37, 65, 83, 84] with noise defined by an adjacent frequency bin [32, 33, 84] or mean of the two adjacent bins [21–23, 26, 65]. Absence of large artefacts at “noise” frequencies has often been used as an additional criterion for accepting presence of a VEP [14, 33, 65]. Criteria based on absolute amplitude or magnitude (e.g. 1 μV) are not reliable because of high interindividual variability in noise and in VEP magnitude [85]. A SNR of 3, based on noise magnitude at one adjacent frequency bin, is associated with an empirical false “positive” rate of 0.3% [33], i.e. a 1-in-333 chance of incorrectly declaring noise to be a VEP. Conversely, a SNR of 3, based on noise as mean of magnitudes at two adjacent bins, has a 4.1% empirical false positive rate [85], i.e. a 1-in-25 chance of incorrectly declaring a VEP to be noise. A SNR of at least 3 therefore appears to represent a suitable criterion with acceptable sensitivity and specificity.

Unless the DFT output at the stimulus frequency is adjusted for noise estimates, SNR is more correctly signal-plus-noise to noise ratio because the signal’s frequency bin also includes noise, i.e. non-visually driven EEG occurring at the stimulus frequency [12, 85]. An alternative criterion requires that the 95% confidence interval of magnitudes, calculated from DFTs of several EEG epochs, should exclude zero: it is not stated whether the signal magnitude measures are noise-corrected (Enfant® proprietary technique, Diopsys Inc., Pine Brook, NJ, USA [18]).

Neither SNR nor magnitude criteria use the phase data produced by Fourier analysis. Phase tends to increase (lag) gradually across coarser SFs and then lag more steeply across higher SF stimuli. It may show large shifts at mid-range SFs, particularly if there is a “notch”. Phase coherence is better to supra-threshold gratings, while noise is characterised by highly incoherent phase [30, 49, 86, 87]. These characteristics have been employed for signal detection alongside SNR criteria, either by requiring physiologically plausible phase lead or lag with decreasing or increasing SF respectively [14, 21, 22, 26, 28, 32, 33, 88], or by requiring that the 95% confidence interval of phase exceeds an empirical criterion of 90° [30, 55].

Fourier analysis produces bivariate data (phase and amplitude); the sine and cosine coefficients can be used to create a complex plane vector for each EEG epoch, with vector length representing magnitude and angle representing phase. Hotelling’s t^2^ statistic [89] and the more powerful circular T^2^ statistic [90] assume a VEP to be present if the elliptical or circular 95% confidence intervals constructed around the vector tips exclude the origin. The circular T^2^ statistic assumes equal variances for the real and imaginary vector components (hence “circular”) and is equivalent to the magnitude-squared coherence statistic [91]. The criterion of 95% confidence interval excluding the origin is identical to a SNR > 1, where signal is defined as mean vector length (VEP magnitude) and noise is defined as radius of the confidence interval [18, 27, 92].

Comparing vectors from stimulated EEG segments with no-stimulus vectors improved VEP SF limits a little compared with a magnitude-only criterion (26.4 cpd vs. 25.4 cpd). A phase-stability criterion produced even better VEP SF limits (30.3 cpd) [20]. These analyses can be used on raw (non-averaged) data, affording real-time analysis [92]. Statistics which use both magnitude and phase outperform those using only one [91]. However, in real-time analyses, different statistics can be complementary. A SNR criterion detected supra-threshold VEPs sooner than the circular T^2^ statistic because it can be applied as soon as the first EEG epoch is acquired, while the circular T^2^ statistic cannot be applied until three EEG epochs are available. Conversely, in low SNR conditions, the circular T^2^ statistic is more powerful and detected VEPs close to threshold faster than the SNR criterion [93]. With suitable adjustment for multiple tests, SNR and circular T^2^ statistic can be used simultaneously to minimise the duration of VEP SF limit testing in a real-time system designed for assessing paediatric patients [92, 94, 95].

For techniques using VEPs (transient or steady state) analysed in the time domain, presence or absence of a VEP is determined subjectively by eye [40, 41, 53, 54, 69, 70, 87, 96–102], sometimes with an additional requirement of a criterion amplitude for P100 [100, 103]. Time domain, objective methods [104–106], which can be employed adaptively in real time to shorten recordings to the minimum necessary for an objective quality measure of the averaged response [107], are unfortunately seldom used for transient VEPs.

d. Definition of threshold

**Fig. 4:**
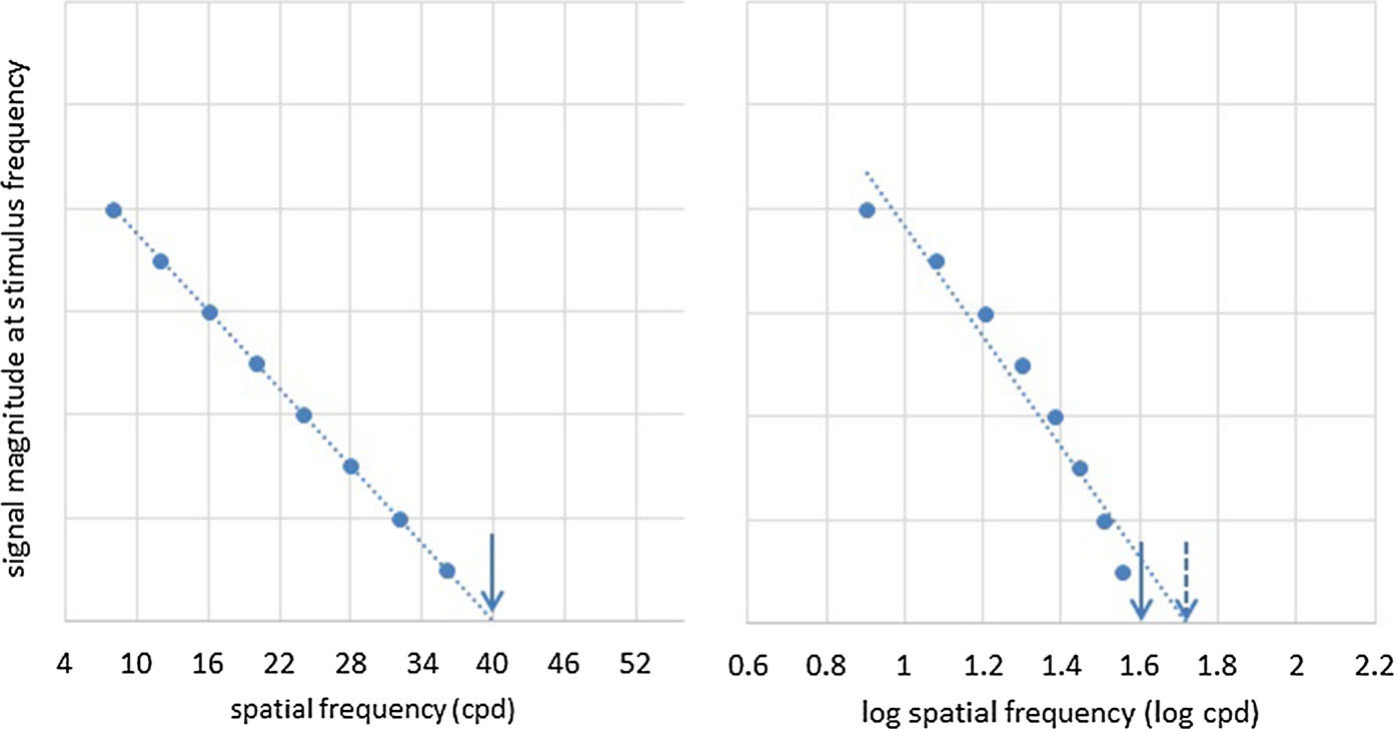
Effect of scaling—illustration of VEP SF limits for artificial data plotted versus linear (left) and versus logarithmic (right) SF. Linear extrapolation to 0 μV with a linear SF axis scale (left) gives a VEP SF limit of 40 cpd (1.6 log cpd) (solid arrows). Linear extrapolation of the same data to 0 μV using a logarithmic SF axis scale (right) gives a “better” VEP SF limit of 50 cpd (1.7 log cpd) (dashed arrow). If the true relationship between VEP amplitude and linear SF close to threshold is linear as shown, then logarithmic SF scaling with linear regression may introduce a systematic error giving erroneously “better” VEP SF limits

The majority of studies define the VEP SF limit by extrapolating a straight line regressed through significant VEP amplitudes or magnitudes plotted versus SF to 0 μV or to another floor such as a noise estimate. The commonly used linear extrapolation to 0 μV approach aimed to minimise bias since the VEP is likely to still be present, but below noise amplitude, at the SFs closest to threshold. It assumes that the function which holds for supra-noise VEPs will also hold for sub-noise VEPs [29]. A few studies define the VEP SF limit by curvilinear fitting, e.g. parabolic, modified Ricker or other curves [78, 98, 102, 108, 109] to magnitude or amplitude data plotted versus SF. These functions are commonly fitted to plots where a linear scale has been used for SF, although logarithmic scales have also been used (Fig. 4) [12, 15–17, 31, 40, 54, 110–113]. A linear SF scale is justified since log contrast sensitivity drops linearly with SF at high SF (> 5 cpd) [114] and VEP amplitude drops linearly with log contrast close to threshold [6, 115]. VEP amplitude therefore theoretically drops linearly with linear SF close to threshold [33]. A linear–linear relationship has been demonstrated in adults [29, 116] and linear extrapolation to zero microvolts on a linear SF axis is insensitive to VEP amplitude changes [117]; logarithmic SF scaling potentially introduces a systematic error, skewing the linear regression to “better” thresholds—the greater the number of points away from threshold used, the greater the skew (Fig. 4).

Certainly, better thresholds with logarithmically scaled SF were found in the two studies which compared VEP SF limits with linear and with logarithmic SF scaling of the same data. Mean VEP SF limits from fourteen eyes of seven healthy individuals were better, but more variable, for logarithmic SF scaling (37 cpd, range 29–73 cpd) than for linear SF scaling of the same data (30 cpd, range 26–41 cpd) [13]. A study of 21 normally sighted adults and older children also found better VEP SF limits for logarithmically scaled SF (median 16 cpd, range 5–243 cpd) than for linearly scaled SF (median 11 cpd, range 4–30 cpd) [84]. Each study found logarithmically scaled SFs produced two cases with unrealistically good limits, e.g. 67 and 73 cpd [13] and 158 and 243 cpd [84]. Both studies used a limited range of SFs with the finest SFs being only 10 or 12 cpd: the larger the gap between the highest SF available and the individual’s VEP SF limit, the larger the error in the extrapolated limit. The error is much larger, and skewed to unrealistically good limits, when logarithmic SF scaling is used.

For the extrapolation limit, multiple criteria have been employed to define threshold, most commonly 0 μV or some estimate of noise floor. Studies comparing the effect of absolute floor levels (0, 1 and 2 μV) found, as might be expected, that VEP SF limits worsened by at least 5 cpd with each 1 μV increase in noise criteria [55, 118]. The 0 μV criterion is widely used, perhaps originating from early experiments where extensive time domain averaging of ssVEPs reduced noise to negligible levels [20], but also theoretically justified since at the point of absent cortical signal, i.e. acuity limit, neuronal noise (as opposed to EEG noise) is low. However, some of the magnitude output of a DFT at the stimulus frequency is due to non-visually driven EEG at that frequency, and hence is noise [85]: the relative proportion which is noise increases in small VEP signal conditions, e.g. close to threshold. Therefore, “raw” magnitudes extrapolated to 0 μV will overestimate VEP SF limits compared with noise-corrected magnitude estimates [12, 25, 44]. This overestimation is likely to be small for signals with good SNR: Norcia et al. note that threshold estimates based on data points with SNR > 3 are “virtually uncontaminated” by EEG noise [33, 85]: at a noise-corrected SNR of 3, there is little (5.3%) noise [85]. If noise estimates are not discarded before extrapolation [85], any overestimation relative to noise-corrected amplitudes could be reduced by extrapolating to a noise floor rather than to zero [20, 27].

An alternative strategy defines VEP SF limit as the finest SF evoking a significant VEP, which ought to underestimate thresholds found by extrapolation. A direct comparison of the two techniques in adults does indeed show an underestimation of 0.5–1 octave (0.15–0.3 log units) [38], or 0.25–0.5 octaves (0.08–0.15 log units) [33], but the underestimate depends strongly on SF sampling density close to threshold. Adult thresholds using the finest SF technique do not differ markedly from other studies reporting thresholds based on extrapolation (see Fig. 2): for example, two studies using the finest SF technique found VEP SF limits of 9.7–40 cpd [19] and 9.4–24 cpd [92], similar to those typically found for extrapolation techniques. No study has compared the two techniques in the same subjects, although retrospective analysis would be straightforward. Since the finest SF technique does not require SF sampling density sufficient to characterise a major portion of the VEP magnitude versus SF function, there is potential for defining limits faster by concentrating recording close to threshold [92].

Extrapolation techniques can fail to define a VEP SF limit, usually because the final, descending limb of the magnitude versus SF function is poorly defined due to deep notches or generally low amplitudes. These failures occurred on 29/384 individual sweeps (8%) [30] and in 2/108 recordings (2%) [12]. One approach under such circumstances is to include the finest SF technique as an additional, integrated strategy [18, 30, 35].

Data acquisition which is time-locked to the stimulus avoids artefacts (overspill or leakage into nearby frequency bins) from the Fourier analysis, thus maximising SNR [27, 119]. This can be achieved by appropriate selection of EEG epoch (i.e. sweep duration or analysis period) as an integer multiple of stimulus periods. For example, an 8.0 Hz stimulus has a period of 125 ms: EEG epochs for analysis should be some integer multiple such as 8 × 125 ms, i.e. 1000 ms. If stimulus and acquisition are not appropriately time-locked, then frequency domain artefacts can be reduced, but not eliminated, by application of windowing techniques. Artefacts can be eliminated by truncating the analysis interval to encompass an integer number of stimulus cycles [119].

In summary, active electrodes close to Oz are sited to define VEP SF limits, and closely positioned reference electrodes, especially in a Laplacian montage, enhance SNR towards threshold. Frequency domain analysis, usually via a DFT, can be subjected to statistical analyses to determine the likelihood of a signal at the stimulus frequency being noise: these statistics can employ magnitude-only measures such as SNR, or include additional phase criteria, or combine magnitude and phase (e.g. circular T^2^). VEP SF limits can be defined by linear (or other functions) extrapolation of significant (non-noise) VEPs plotted versus linearly or logarithmically scaled SF: logarithmic SF scaling may introduce a systematic error, skewing the linear regression to better thresholds. The intercept with 0 μV is commonly used to define the VEP SF limit; this may result in slightly better thresholds for “raw” VEP magnitudes compared with noise-corrected VEP magnitudes. Alternative noise floors based on measured levels avoids this overestimation for non-noise-corrected DFT magnitudes. Extrapolation techniques occasionally fail to define a VEP SF limit. An alternative, and possibly faster, approach to extrapolation is to use the finest SF criteria to define VEP SF limit. Optimal, artefact-free EEG spectra can be ensured by using an EEG sampling rate which is an integer multiple of the monitor’s frame rate, if relevant, and by choosing or truncating the analysis period to be an integer multiple of the stimulus period.

### Correspondence of VEP SF limits with behavioural thresholds in normally sighted adults

In some of the work already described, an implicit or explicit aim was to develop a VEP technique whose SF limit agreed with perceptual thresholds. Often, close agreement was taken as an indication of the quality of the VEP technique, even though an exact match would be surprising given the multiple different mechanisms involved, listed in the introduction. Agreement has sometimes been “improved” using techniques which may have some systematic error or bias, for example logarithmic SF scaling or using a 0 μV intercept for non-noise-corrected magnitudes. The aim of this section is to describe disparities between VEP SF limits and perceptual SF thresholds under three circumstances: studies employing identical stimuli; studies comparing VEP SF limits with behavioural acuity tests using discrimination tasks; studies comparing VEP SF limits with behavioural acuity tests using identification tasks.

#### Identical stimuli

**Fig. 5:**
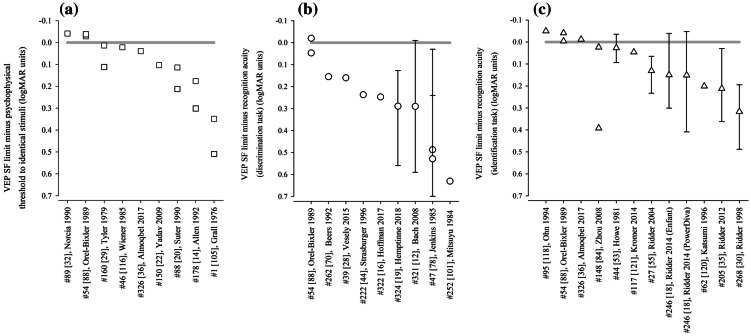
Illustration of studies reporting differences between VEP SF limits and behavioural thresholds in healthy adults using **a** psychophysical testing with VEP stimuli (Table 2); **b** recognition acuity based on a discrimination task (Table 3) and **c** recognition acuity based on an identification task (Table 4). Grey lines indicate no difference: points below the line indicate VEP SF limits are poorer than behavioural thresholds. Error bars indicate 95% limits of agreement (not always available)

We identified nine studies where the same, normally sighted adult subjects had psychophysical acuity and VEP SF limits assessed using identical stimuli (Table 2, Fig. 5a). Seven found poorer VEP SF limits than psychophysical acuity. An early paper recorded three thresholds to a red and black reversing checkerboard by changing the viewing distance: subjects could perceive apparent motion at an average distance of 4.2 m (≈ 10.2 cpd), could perceive stationary checkerboards at 2.9 m (≈ 7.05 cpd) and could evoke measurable VEPs at around 1.3 m (≈ 3.2 cpd) [105]. VEP SF limits in four adults were about 25 cpd, while psychophysical thresholds, based on two-alternative forced-choice technique, were about 50 cpd for high-luminance (100 cd s/m^2^) gratings—the difference lessened for dimmer gratings [14]. Based on a button press at the end of each VEP trial if a grating had been seen, psychophysical thresholds (42.5 cpd) exceeded VEP SF limits using either magnitude (26.1 cpd) or phase (32.7 cpd) criteria [20]. Similarly, psychophysical thresholds were 42.5 and 42.4 cpd on average compared with mean VEP SF limits of around 33.5 and 38.7 cpd [22, 36]. Sweep VEP SF limits to reversing sinewave gratings were 25 cpd on average, slightly poorer than psychophysical thresholds of 26.3 cpd [116]. In a study of a single adult, an 11.3 cpd VEP SF limit and a 14.6 cpd psychophysical threshold improved to 31 cpd and 32 cpd, respectively, by increasing mean luminance from 46 to 360 cd m^−2^, reducing field size from 20 × 15° to 2 × 2° and increasing contrast from 80 to 90% [29]; Nelson et al. subsequently noted of this study that VEP SF limits averaged 85% of psychophysical acuity limits [25]. In two studies using very similar methodologies, the reverse situation was found: VEP SF limits were slightly better than psychophysical thresholds (31.9 vs. 29.0 cpd [32], 37.5 vs. 35.1 cpd [88]).

**Table 2:** Comparison of average VEP SF limits and psychophysical thresholds in normally sighted adults using identical stimuli (see Fig. 5a)

Study	Number of subjects	VEP stimulus	VEP SF limit (cpd)	Psychophysical threshold (cpd)	VEP minus psychophysical difference (log units)	VEP method	Psychophysical method
#54 [88]	16	Vertical sinusoidal gratings, 80%, 80 cd m^−2^, 15 rps, coarse-to-fine	37.5	35.1	− 0.029	Monocular, Oz–O1/O2, DFT, linear extrapolation of magnitude to 0 μV	Monocular, method of ascending and descending limits, button press to indicate grating appearance/disappearance, geometric mean of 5 trials
		as above but fine-to-coarse	36.3	33.3	− 0.038		
#326 [36]	16	Horizontal sinusoidal gratings, 90%, 50 cd m^−2^, 15 rps, coarse-to-fine	38.7 (SEM 1.2)	42.4 (SEM 1.2)	0.039	Binocular, 5 occipital channels, DFT, linear extrapolation of magnitude to 0 μV	Temporal, 2-alternative forced-choice, 2-down, 1-up staircase, last 10 measurements for threshold: 77% correct
#88 [20]	10	Vertical sinusoidal gratings, 40%, 40 cd m^−2^, 12 or 7.5 rps, 2 × 3°	26.1 (mag) 32.7 (phase)	42.5	0.212 0.114	Oz–RO/LO, DFT, linear extrapolation of magnitude or phase versus SF to noise floor	Simultaneous button press at end of each VEP trial if a grating was seen; 50% threshold
#46 [116]	8	Monocular, vertical and horizontal sinusoidal gratings, 86%, 50 cd m^−2^, 7 rps, 2 × 3°	25	26.3	0.022	Oz–earlobe, sweep, linear extrapolation of amplitude to amplifier baseline	Verbal indication when moving sensation first occurred; method of adjustment, average of 2 ascending and 2 descending limits
#89 [32]	7; 10	Vertical sinusoidal gratings, 80%, 220 cd m^−2^, 12 rps, 14 × 28 cm	31.9	29	− 0.041	Oz–O1/O2, DFT, linear extrapolation of magnitude to 0 μV	Psychophysical CS function including 80%; ascending and descending limits, button push at threshold
#150 [22]	6	Horizontal sinusoidal gratings, 40%, 50 cd m^−2^, 15 rps, 6.3 × 6°	33.5 (SD 4.5)	42.5 (SD 2.6)	0.103	Oz–Cz, adaptive filter, linear extrapolation of magnitude to 0 μV (automated, “C0”)	2-alternative FC staircase, 2-down, 1-up, converge to 82% threshold, 35–60 cpd, step size 1/10 of range
#178 [14]	4	Vertical sinusoidal gratings, 80%, 0.1 cd m^−2^, 12 rps, @ 80 cm	2.8	4.2	0.176	Oz–lateral, DFT, linear extrapolation of magnitude to 0 μV	Forced-choice 2-alternative technique using same stimulus at 80 cm, 3–5 SFs, 75% correct
		as above but 100 cd m^−2^	25	50	0.301		
#160 [29]	1	Vertical sinusoidal gratings, 80%, 46 cd m^−2^, 24 rps, 20 × 15°	11.3	14.6	0.111	Oz–lateral, synchronous filter, linear extrapolation of relative amplitude to 0 μV	Psychophysical version of VEP stimulus, methods of adjustment, observer adjusted SF until threshold × 3
		as above but square-wave gratings, 90%, 360 cd m^−2^, 2 × 2°	31	32	0.014		
#1 [105]	not stated	Red (lit) and black (off) 14 × 7 photodiode checkerboard, 2 rps, 36 × 18 mm	≈ 3.2	≈ 7.05 or ≈ 10.2	0.349 or 0.503	Iz–mastoid, objective time domain analysis, distance at which VEP not significant	Subjects indicate the distance from which the checkerboard phase reversal is no longer perceived

Hashtag numbers indicate the 155 references whose data were included in the systematic review

These data suggest that VEP SF limits are per se different to, and probably slightly poorer than, behavioural or psychophysical thresholds to identical stimuli. The gap is very variable across the studies reviewed and depends strongly on the stimulus, with high contrast, high-luminance stimuli generally producing closer agreement than for lower contrast, lower luminance stimuli (Fig. 5a).

#### Non-identical stimuli—recognition (discrimination) acuity tests

We identified nine studies where the normally sighted adult subjects had both VEP SF limits and a discrimination acuity measured, i.e. tumbling E or Landolt C charts (Table 3, Fig. 5b). Studies were included if sufficient detail was available to describe both average VEP SF limits and acuities: when possible, variability and limits of agreement were also extracted. Often, these findings were part of a study which included a spectrum of visual abilities, with poorer vision recorded from patients or from artificially impaired adults: only data from healthy, optimally refracted adults are presented in this section. Acuities were converted from native units into logMAR units as necessary, and VEP SF limits (cpd) were also expressed in logMAR units to allow comparison with acuity (Table 1).

**Table 3:** Comparison of average VEP SF limits and behavioural acuity (recognition based on a discrimination task) in normally sighted adults

Study	Number of subjects	VEP stimulus	Average VEP SF limit	Average acuity (detection task)	VEP minus behavioural difference (log units) 95% LoA	VEP method	Discrimination acuity method
#321 [12]	40	On/offset (40/93 ms, 7.52 Hz) checkerboard, 40%, 45 cd m^−2^	26.7 cpd 0.051 logMAR	− 0.241 logMAR	0.290 − 0.010 to 0.590	Monocular, Laplacian around Oz, DFT, automated extrapolation of significant, noise-corrected magnitudes versus log SF to 0 μV	Freiburg acuity test (FrACT) with Landolt Cs, monocular
#47 [78]	16	Reversing (6 rps) checkerboard, 88%, 72 cd m^−2^, 8.4 × 6.5°–16.8 × 13°	16.2 cpd 0.297 logMAR	− 0.211 logMAR	0.508 0.278 to 0.693	Monocular, Oz–Fpz, time domain and frequency domain, extrapolated curvilinear or linear amplitude versus checksize	Landolt C bracketed “walk-back”, forced-choice technique, 8/10 correct, 246 cd m^−2^, 79%, monocular, repeated × 3
#54 [88]	16	Vertical sinusoidal gratings, 80%, 80 cd m^−2^, 15 rps	37.5 cpd − 0.097 logMAR	− 0.077 logMAR	− 0.020	Monocular, Oz–O1/O2, DFT, linear extrapolation of magnitude to 0 μV	Projected tumbling Es, descending method of limits, 4 alternative forced-choice, monocular
				− 0.144 logMAR	0.047		Flom S-chart, Landolt Cs and tumbling Es, 4 alternative forced-choice, 50% correct from psychometric function, monocular
#262 [70]	13	on/offset (40/400 ms, 2.3 Hz) checkerboard, “350 lx”, 2 × 2°	24.8 cpd 0.083 logMAR	≤ 0.000 logMAR	“Typically 0.155″	Monocular, Laplacian around Oz, 50 averages, subjectively judged reproducibility, amplitude versus checksize plot, linear regression through “clear descending trend” to 0 μV	Landolt C chart at 5 m, 350 lx, monocular
#324 [19]	13	Vertical sinusoidal gratings, reversing (20 rps), 30%, 132 cd m^−2^, 20 × 20°	21.8 cpd 0.135 logMAR	− 0.162 logMAR	0.289 0.127 to 0.559	Monocular, optimal electrode from 64-channel array, time domain averaging–DFT, minimal SF with significant, noise-corrected magnitude	FrACT logMAR tumbling Es, monocular
#252 [101]	9	Reversing (2 rps) checkerboard, 20 or 40%, 42 or 10 cd m^−2^, 4.5 × 4.5°	~7 cpd ~0.63 logMAR	0.000 logMAR	0.63	Monocular, Oz–earlobe, time domain averaging, minimal SF with subjectively judged VEP	Landolt test chart, monocular (data extrapolated only for subjects with decimal acuity 1.0)
#322 [16]	2	On/offset (40/93 ms, 7.52 Hz) checkerboard, 40%, 45 cd m^−2^	26 cpd 0.062 logMAR	− 0.182 logMAR	0.247	Monocular, Laplacian around Oz, DFT, automated extrapolation of significant, noise-corrected magnitudes versus log SF to 0 μV	Uncrowded FrACT, Landolt Cs, monocular, repeated
#39 [28]	1	Reversing (12 rps) checkerboard, 60%, 80 cd m^−2^	19.37 cpd 0.190 logMAR	0.027 logMAR	0.160	Monocular Oz–Fz, DFT, linear extrapolation of magnitude versus SF to 0 μV	Landolt C chart, monocular
#222 [44]	1	Vertical sinusoidal gratings, on/off (sinusoidal, 16 Hz), 40%, 17 cd m^−2^, 5 × 5°	20 cpd 0.176 logMAR	− 0.06 logMAR	0.237	Oz–Cz, time domain averaging–DFT–vector averaging, extrapolation	Landolt acuity

LoA: 95% limits of agreement (see Fig. 5b). Hashtag numbers indicate the 155 references whose data were included in the systematic review

Average acuities were better than VEP SF limits in most cases. Extrapolating findings for nine subjects performing at the 0.000 logMAR level showed average VEP SF limits around 7 cpd (checkwidth subtending about 6′), a 0.63 log unit difference: use of transient VEPs and few pattern sizes near threshold may have resulted in subject fatigue or a floor effect causing this relatively large difference [101]. A study of 16 adults with a very thorough psychophysical acuity method also found a large VEP-psychophysical difference of 0.529 log units based on time domain analysis of steady-state VEPs, and a slightly smaller difference of 0.487 log units based on frequency domain analysis of the same data [78]. Two larger studies with 40 and 13 subjects, respectively, had very similar differences between VEP SF limits and acuity, of 0.290 and 0.289 log units despite methodological differences [12, 19]. Studies describing only one or two normally sighted adult subjects found similar VEP SF limits (19.37, 20 and 25 cpd, i.e. 0.190, 0.176 and 0.082 logMAR) for average acuities of 0.027, − 0.06 and − 0.182 logMAR, respectively, and hence VEP—behavioural differences of 0.163, 0.236 and 0.264 log units, respectively [16, 28, 44]. The difference was “typically” 0.155 log units in a study which noted acuities of “at least” 0.000 logMAR [70]. Eight of the nine studies found better discrimination acuity than VEP SF limits, with the difference ranging from around 0.15 to 0.6 log units (Fig. 5b). One study found very similar acuity and VEP SF limits using a VEP technique which also produced VEP SF limits closely matched to psychophysical thresholds for the VEP stimulus [88].

Three studies presented limits of agreement between behavioural acuity and VEP limit, or these were calculable from tabulated data: limits were wide: ± 0.2–0.3 log units [12, 19, 78]. Together, these data suggest that, while VEP SF limits can be close to recognition (discrimination) acuity, the difference is very variable and depends strongly on the combination of VEP technique (stimulus and analysis) and acuity technique used.

#### Non-identical stimuli—recognition (identification) acuity tests

We identified eleven studies where normally sighted adult subjects had both VEP SF thresholds measured and also a behavioural acuity test based on an identification task, i.e. one of the many letter charts (Table 4, Fig. 5c). Again, studies were only included if sufficient detail was available to describe both average VEP SF limits and behavioural acuity: when possible, limits of agreement were also extracted. Acuities were converted from native units into logMAR units as necessary, and VEP SF limits were expressed in logMAR units to allow comparison with acuities.

**Table 4:** Comparison of average VEP SF limits and behavioural acuity (recognition based on an identification task) in normally sighted adults

Study	Number of subjects	VEP stimulus	Average VEP SF limit (logMAR)	Detection acuity (logMAR)	VEP minus behavioural difference (log units) 95% LoA	VEP method	Recognition acuity method
#95 [118]	42	Checkerboard, 30%, 50 cd m^−2^, 12rps, 10 × 10°	− 0.088	− 0.039	− 0.049	Monocular, Oz–Fz, subjective time domain analysis, linear extrapolation of linear portion of amplitude versus checkwidth plot to 0 μV	Retro-illuminated ETDRS chart, monocular
#117 [121]	33	Horizontal sinusoidal gratings	− 0.076	− 0.122	0.046	Monocular, Oz–Fz; “automated results”	ETDRS 2000 chart, monocular
#246 [18]	25	Horizontal sinusoidal gratings, 15rps, 80%, 100 cd m^−2^, 13 × 10°	0.064	− 0.086	0.150 − 0.039 to 0.301	Monocular, Oz–earlobe, DFT, significant points’ magnitude 95% CI excludes zero, magnitude versus SF plot, linear regression through significant data to 0 μV, or finest SF	ETDRS, monocular
		as above but 7 × 6°	0.065		0.151 − 0.047 to 0.409	As above, but significant points had SNR ≥ 1	
#205 [35]	24	Horizontal sinusoidal gratings, 15rps, 80%, 100 cd m^−2^, 13 × 10°	0.148	− 0.164	0.212 0.003 to 0.362	Binocular, Oz–earlobe, DFT, significant points’ magnitude/phase 95% CI excludes zero/< 90°, magnitude versus SF plot, linear regression through significant data to 0 μV, or finest SF	logMAR EDTRS, binocular
#54 [88]	16	Vertical sinusoidal gratings, 80%, 80 cd m^−2^, 15rps	− 0.097	− 0.093	− 0.004	Monocular, Oz–O1/O2, DFT, linear extrapolation of magnitude to 0 μV	AO letter chart
				− 0.056	− 0.041		Bailey–Lovie chart
#268 [30]	16	Horizontal sinusoidal gratings, 15rps, 80%, 100 cd m^−2^, 19 × 15°	0.200	− 0.117	0.317 0.195 to 0.488	Binocular, Oz–earlobe, DFT, significant points’ magnitude/phase 95% CI excludes zero/< 90°, magnitude versus SF plot, linear regression through significant data to 0 μV, or finest SF	Bailey–Lovie chart, monocular
#326 [36]	16	Horizontal sinusoidal gratings, 90%, 50 cd m^−2^, 15rps	− 0.111	− 0.099	− 0.012	Binocular, 5 occipital channels, DTF, linear extrapolation of magnitude to 0 μV	Bailey–Lovie crowded letter chart
#44 [53]	12	On/offset (40/460 ms, 2 Hz) checkerboard, 95%, 10 cd m^−2^, 5 × 4°	− 0.021	− 0.048	0.027 − 0.035 to 0.094	Oz–Fz, transient VEPs, CI–CII versus log contrast for each SF, linear extrapolation to 0 μV for threshold; log contrast threshold versus log checkwidth, linear extrapolation to 100% contrast.	Snellen chart
#27 [55]	10	Horizontal sinusoidal gratings, 15rps, 80%, 100 cd m^−2^, 13 × 10°	0.075	− 0.056	0.131 0.056 to 0.233	Binocular, Oz–earlobe, ≥ 7 coarse-to-fine sweeps, DFT, significance SNR > 2 relative to 1 neighbour bin, amplitude versus log SF plot, linear regression through significant data to 0 μV	Bailey–Lovie chart 4, binocular
#62 [120]	6	Vertical sinusoidal gratings, 90%, 12rps, 14 × 14°	0.143	− 0.058	0.201	Monocular, Oz–Pz, DFT, linear extrapolation of linear portion of magnitude versus log SF function to 0 μV	Retro-illuminated ETDRS chart, monocular
#148 [84]	3	Horizontal sinusoidal gratings, 97%, 100 cd m^−2^, 12rps, 23 × 17°	− 0.109	− 0.133	0.024	Monocular, Oz–Fz, DFT, linear extrapolation of linear portion of significant magnitude versus log visual angle to 0 μV	Bailey–Lovie chart
			0.259		0.392	As above, but extrapolated magnitude versus linear SF plot	

LoA: 95% limits of agreement (see Fig. 5c). Hashtag numbers indicate the 155 references whose data were included in the systematic review

Eight of the 11 studies found average recognition (identification) acuity to be better than VEP SF limits. The offset ranged from around 0.03 to 0.3 log units, a smaller difference than for VEP SF limits versus discrimination acuity (Fig. 5b) and similar to the differences found using psychophysical testing with stimuli identical to VEP stimuli. The largest difference, 0.317 log units, may be due to the relatively small number (*N* = 6) of SFs used [30]. Four of these eight studies found relatively small offsets of 0.1–0.2 logMAR using relative high-luminance, high-contrast and large field VEP stimuli and both logMAR and Snellen standard clinical letter charts [18, 35, 55, 120], and three studies found offsets of < 0.1 log units despite widely varying techniques [53, 84, 121]. Finally, three studies found better VEP SF thresholds than behavioural acuities. Two used high-contrast gratings, multiple SFs and objective frequency domain analysis [36, 88], while the other used low-contrast checkerboards and subjective time domain analysis with a finest SF of only 4.2 cpd, but extrapolated versus logarithmically scaled SF [118].

Several studies tabulated data, allowing 95% limits of agreement to be calculated: these were around ± 0.05–0.25 log units, somewhat narrower limits than found for VEP versus discrimination acuity tasks.

These findings for normally sighted adults indicate that it is usual for psychophysical or clinical acuity measures to be better than VEP SF limits. For this reason, inferring a behavioural acuity of 0.000 logMAR because of a VEP SF limit of 30 cpd in adults is very likely to underestimate behavioural acuity: in general, attributing a behavioural acuity which is the geometric equivalent of the VEP SF limit to an individual will be incorrect. An empirical calibration factor (additive offset on a log scale) is required before inferring behavioural acuities from a VEP SF limit: the value of this offset is highly variable and strongly dependent on both the VEP stimulus and analysis process and on the behavioural test (Fig. 5). The value of the offset for adults has been derived in detail in some instances, e.g. 17.6 deg^−1^ (0.232 logMAR) over a wide range of acuity [12], but is unlikely to apply to different combinations of VEP and behavioural acuity test protocols. Theoretical reasons for the offset, outlined in the introduction, are based on the different requirements of a VEP task and a psychophysical acuity test, including differences in neural substrates, inherent SNR, thresholding technique and stimulus properties.

A small VEP–behavioural offset is not necessarily a desirable goal: any offset can be handled by a calibration factor or allowed for in clinical interpretation of the VEP SF limit. A likely greater obstacle for clinical use of VEP SF limits as a proxy for acuity is the width of the limits of agreement generally found for VEP SF limits and behavioural acuity (Fig. 5b, c).

### VEP SF limits in typically developing infants and children

VEP estimation of acuity is a critical tool in paediatric vision testing, particularly where co-morbidities such as cerebral palsy (CP), cerebral visual impairment (CVI) or eye movement disorders reduce the usefulness of conventional clinical acuity tests such as matching recognition tests or acuity card tests based on fixation preference. Paediatric clinics also require tools to assess children suspected of having non-organic vision loss (NOVL) and VEP estimation of acuity can be useful in this role. The utility of VEP SF limits for all clinical applications is heavily dependent on a detailed understanding of what is “normal”, i.e. the range of VEP SF limits in typically developing infants and children.

#### Effect of stimulus, acquisition and analysis variables on VEP SF limits of typically developing infants and children

Compared with adult studies, relative few workers have explored the effects of stimulus, acquisition and threshold estimation techniques in children. In general, VEP techniques optimised for adults have been modified, e.g. by altering the SF range used, to be useful for testing infants and children.

Some studies have investigated the temporal effects of stimuli, which is relevant due to the potentially confounding effect of maturation of temporal tuning on maturation of spatial tuning, i.e. acuity development [39]. However, no reversal rate effect on VEP SF limits was found for 10, 14 and 24 rps recorded from 42 infants ranging from 2 to 13 months old [122], nor for 12 and 20 rps in 4 and 6 month infants [123], nor for 6 and 8 rps in 10–39-week-old infants [23]. Similarly, changing reversal rates from 12 to 15 to 20 rps did not affect VEP SF limits in 6–8-year-old children [37]. Orel-Bixler and Norcia compared VEP SF limits over the first 6 months of life for two different stimuli: transient, brief on/offset patterns of five SFs and steady-state reversing patterns of 19 SFs: for the youngest subjects, VEP SF limits were better with the steady-state stimuli; thereafter, VEP SF limits converged, matching by around 3–4 months of age, and agreeing quite closely up until 6 months of age [124]. In a study designed to investigate whether the infant retina generates high SF distortion products which evoke VEPs, VEP SF limits were slightly better with brief on/offset 5.5 Hz stimuli than reversal (11 rps) stimuli (8.8 vs. 6.7 cpd) in 18 infants aged 6–17 weeks [125].

VEP SF limits in 15–20-week-old infants improved nonlinearly as stimulus mean luminance increased from 0.01 (≈ 2.5 cpd) to 100 cd m^−2^ (≈ 7 cpd); most improvement occurred between 0.01 and 1 cd m^−2^ [14]. VEP SF limits did not differ in 6–8-year-old children across mean stimulus luminances of 25, 50 and 100 cd m^−2^ [22]. Similarly, typically developing children aged 3–12 years had similar VEP SF limits to low luminance stimuli (14–35 cpd, 20 cd m^−2^) as those to high-luminance stimuli (13–31 cpd, 109 cd m^−2^) [126].

It was noted that children were more attentive to coarse-to-fine SF changes than fine-to-coarse, but there were no significant differences in VEP SF limit with direction of SF change [88]. Almoqbel et al. found better VEP SF limits with coarse-to-fine than fine-to-coarse SF changes (38 vs. 31 cpd) in a small group of 6–8-year-old children: this difference disappeared when a fixation mark was used [37]. The same study found neither field size (2, 4 or 6°) nor stepwise sweep duration (10, 15 or 20 s) to have a significant effect on VEP SF limits. No other studies were found which investigated stimulus effects in typically developing infants and children.

A large developmental study over the first year of life showed binocular VEP SF limits to be slightly better than monocular limits at all ages by < 0.06 log units (< 0.2 octaves), with a trend for the difference to lessen with age. The binocular and monocular maturation curves were very similar, and the binocular–monocular difference was markedly smaller than quoted for behavioural acuity (0.18–0.3 log units, i.e. 0.6–1 octaves) [127]. Similarly, for a small group of children under 5 years of age, average VEP SF limit was 24.3 cpd with binocular viewing, and only slightly poorer (22.9 cpd: a difference of 0.03 log units) with monocular viewing [88].

Infant and child studies have employed one channel or more channels in approximately equal proportion. For two or more channels, data from whichever channel or channel combination gives the “best” results tends to be used. Most commonly, an active electrode at Oz and/or electrodes symmetrically and laterally positioned close to Oz, for example at O1 and O2, or PO7 and PO8, are used. Reference sites are either distant, e.g. Cz, Fpz or earlobe, or near, e.g. O1 and O2 active sites referenced to Oz. A one-dimensional Laplacian montage was shown to detect ssVEPs more often and a few seconds faster than an Oz–Fz electrode montage in children older than about 5 years, although VEP SF limits were not recorded [94].

#### Effect of age on VEP SF limit

As for behavioural acuity, VEP SF limits show marked maturation effects. VEP SF limits are popular as a biomarker for brain development in studies of infant nutrition, thus there is a large body of data describing VEP SF limits in typically developing infants across all nutrition groups. Additionally, many diverse studies of pathology include data from control groups of typically developing children.

**Fig. 6:**
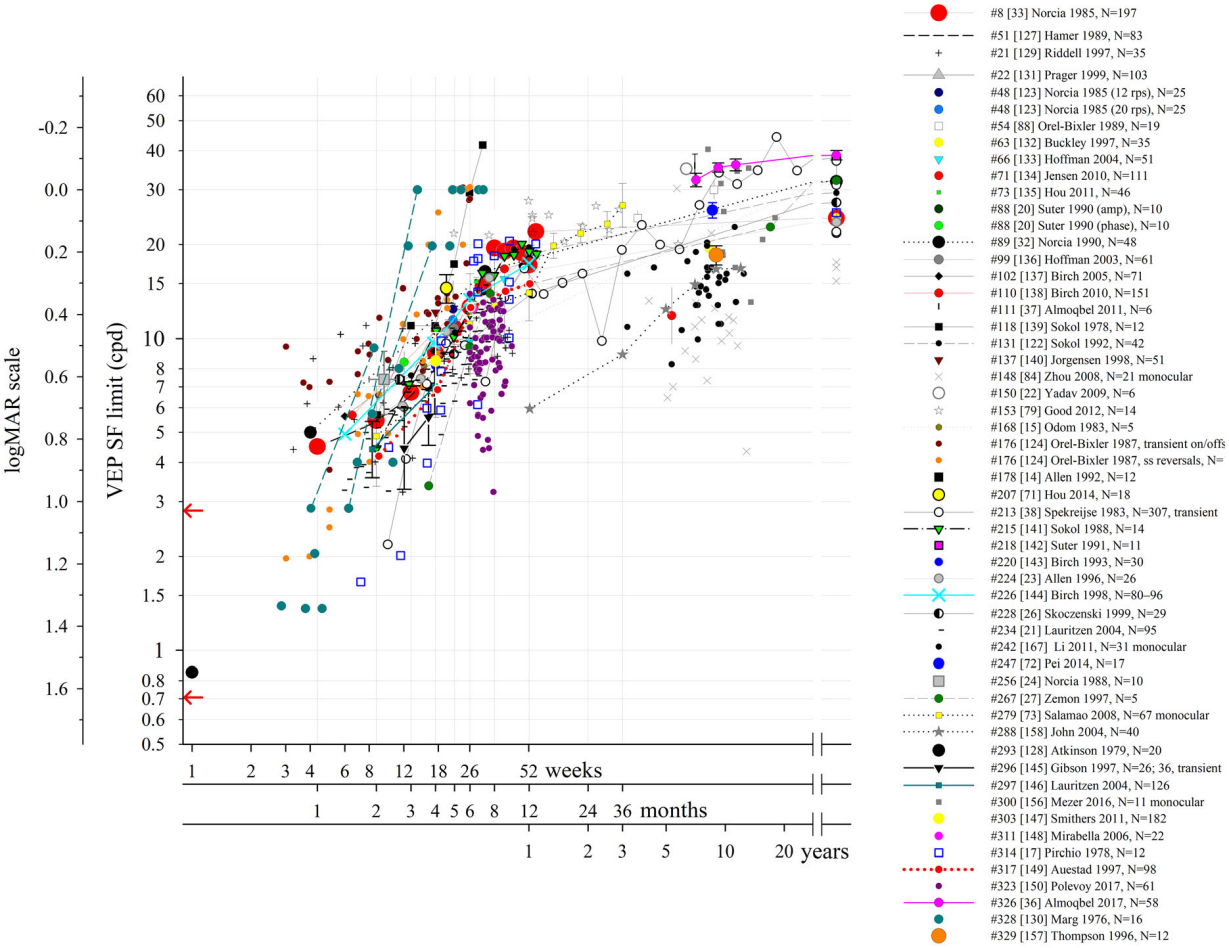
Illustration of the development of VEP SF limits through infancy and childhood from 52 studies. A variety of techniques were employed (see text for details). Dashed lines indicate subjects from a cross-sectional study. Solid lines indicate participants in a longitudinal study. Error bars typically indicate SEM. Red arrows at the SF axis indicate the two ISCEV standard checkwidths, 60′ and 15′ (0.71 and 2.8 cpd)

We identified 52 studies which described VEP SF limits from infants and/or children screened or understood to be typically developing, and born at full-term. For nutritional studies, sub-groups were combined where possible to reflect the typical population. Extracted data were expressed in units of cpd (Fig. 6). The few data available in the first month of life suggest a rapid improvement in VEP SF limit from poorer than 1 cpd in the first few days [128] up to 1.5–9 cpd by the end of the first month [32, 33, 124, 129, 130]. As evident in Fig. 6, there is rapid improvement until 8–12 months when VEP SF limits are typically 15–20 cpd [14, 15, 17, 20, 21, 23, 24, 26, 27, 32, 33, 38, 71, 73, 122–124, 127, 129–154]. A control group of 27 infants aged 6–25 mo had a mean VEP SF limit of 13.4 cpd with a trend towards better limits with age [155].

From 1 year through to adulthood a slower improvement is evident, from 12 to 20 cpd up to 20–40 cpd [17, 22, 26, 27, 32, 33, 37, 38, 72, 73, 88, 132, 156, 157].

In some cases, these values or trends were not observed. Riddell et al. tested 35 infants aged from 1 to 10 months, and found good VEP SF limits from the youngest infants (< 4 mo, 4–11 cpd) with little improvement for the oldest infants (8–10 months, 8–11 cpd), giving a flatter developmental curve [129]. Data from Sokol et al. followed an underlying trend of improvement for ages 2–5 mo, but at 6 and at 7 months, infants had excellent and adult-like VEP SF limits (29 and 42 cpd, respectively), although only nine and two subjects, respectively, contributed to these averages [139]. Similarly, three subjects aged 5–6 months had adult-like VEP SF limits of around 30 cpd [124] and eight subjects aged 3–7 months had adult-like limits of 20 or 30 cpd [130]. In a cross-sectional study of 61 infants aged 6–9 months, there was a large spread of VEP SF limits (3–14 cpd) but no age-related trend [150].

Poorer VEP SF limits than those described above, by around 10 cpd, were reported for 40 control children aged 1–13 years, perhaps partly due to a long study protocol which included VEP contrast limits, behavioural acuity assessment, retinoscopy and accommodation assessment [158]. A large group of 55 children aged 5 years had average VEP SF limits of around 12 cpd [134], at the lower limit of typical limits (15 cpd or better) reported for similar ages [37, 72, 73, 79, 88, 132]: again, children undertook a lengthy protocol including anthropometric, neurodevelopment and multiple vision assessments.

Despite these examples, the majority of studies illustrate a trend of rapid improvement from 1.5 to 9 cpd at 1 month to 12–20 cpd by 8–12 months, followed by slower improvement to 20–40 cpd by adulthood. This consistency may be partly because a majority of paediatric studies use versions of the same stepwise sweep VEP technique developed by Tyler, Norcia and colleagues [29, 159]. However, VEP SF limits established using quite different techniques, for example on/offset transient VEPs, show a very similar developmental curve [38].

As with all forms of paediatric testing, success rates for establishing a VEP SF limit vary with age. Success could be defined as collection of sufficient and adequate data to define a VEP SF limit. Some studies of typically developing infants and children report high success rates, such as 83/87 (95%) [127] and 197/215 (92%) [33] in infants around the first year of life: failures occur in the youngest and oldest subjects. Similarly, data were recorded from 142/147 (97%) 2-month-old infants, and VEP SF limits defined for 126 of the 147 (89%): this improved to 147/148 (99%) and 147/148 (99%) for the same subjects at 4 months [146]. This improved success may have been due to increased infant maturity, interest and/or cooperation, but also to increased parent familiarity with the procedure, or improved skill of the researchers [146]. Other studies report lower success rates, such as data recorded from 48/52 (93%) 12-week-old infants but VEP SF limits defined for only 26/52 (50%): again, this improved to 52/52 and 36/52 (69%) for the same subjects at 16 weeks [145]. All 44 older children (3 months to 14 years) had data successfully collected, but VEP SF limits were defined for only 40/44 (91%) [158]. Children can be particularly erratic in compliance around 2–4 years, and VEP SF limits drop markedly in this age range in a large cross-sectional study, suggesting that compliance not only affects success in obtaining a VEP SF limit, but perhaps also the limit itself [38].

#### Effect of premature birth on VEP SF limit

The effect of premature birth has been investigated by assessing prematurely born infants without sequelae such as retinopathy of prematurity or cerebral injuries, or only mild forms of these, often termed “healthy” preterm infants. One study found slightly but significantly better VEP SF limits (0.14 log units) in 13 healthy infants born prematurely (31.4 (± 3.3 SD) weeks gestation) and tested at post-term ages of 1–10 months, relative to term-born, age-matched controls. Comparison by post-natal age showed no significant difference, suggesting VEP SF limits develop from birth, and therefore that premature birth might accelerate development [160]. No other study with term-born, age-matched controls found this accelerated development; however, 17 infants born prematurely (at 27–32 weeks gestation) and tested at 4 weeks preterm (36 weeks post conception) and at 17 weeks post-term age had VEP SF limits of 4.4 and 13.7 cpd, respectively [161], which are towards the upper end of values reported for term-born infants (Fig. 6). In contrast, four studies each with a term-born, age-matched control group found similar or slightly poorer VEP SF limits in healthy prematurely born infants. Prematurely born versus term-born infant VEP SF limits were 12.4 versus 12.5 cpd [148] and 12.7 versus 15.2 cpd at 6 months corrected age [135]. Two nutritional studies found very similar VEP SF limits (around 8 cpd) in healthy, prematurely born and in term-born control infants at 4 months corrected age [143, 162].

Several other nutritional studies recorded VEP SF limits in healthy, prematurely born infants: these study designs did not include a control group of term-born infants, but limits fitted well within the values collated from typically developing, term-born infants (Fig. 6). Average VEP SF limits for healthy prematurely born infants have been reported as 5.6 cpd at 2 months post-term [163]; 7.4, 8.5 and 8.9 cpd at 4 months post-term ages [83, 163, 164] and 8.9, 11.5 and 13.1 cpd at 6 months post-term [83, 164, 165]. For a large cross-sectional cohort of low- and high-risk prematurely born infants assessed using transient VEPs, VEP SF limits improved from around 2 cpd at term age to around 3.3 cpd at 10 weeks post-term and around 6 cpd at 2 years post-term [166], suggesting a flatter developmental curve than seen in typically developing term-born infants (Fig. 6).

Collectively, these findings suggest that prematurely born infants who largely escape ophthalmic or neurological complications of prematurity are likely to have similar VEP SF limits to their term-born peers, making any adjustment for prematurity unnecessary for healthy preterm infants.

### Correspondence of VEP SF limits with behavioural thresholds in typically developing infants and children

#### Identical stimuli

**Fig. 7:**
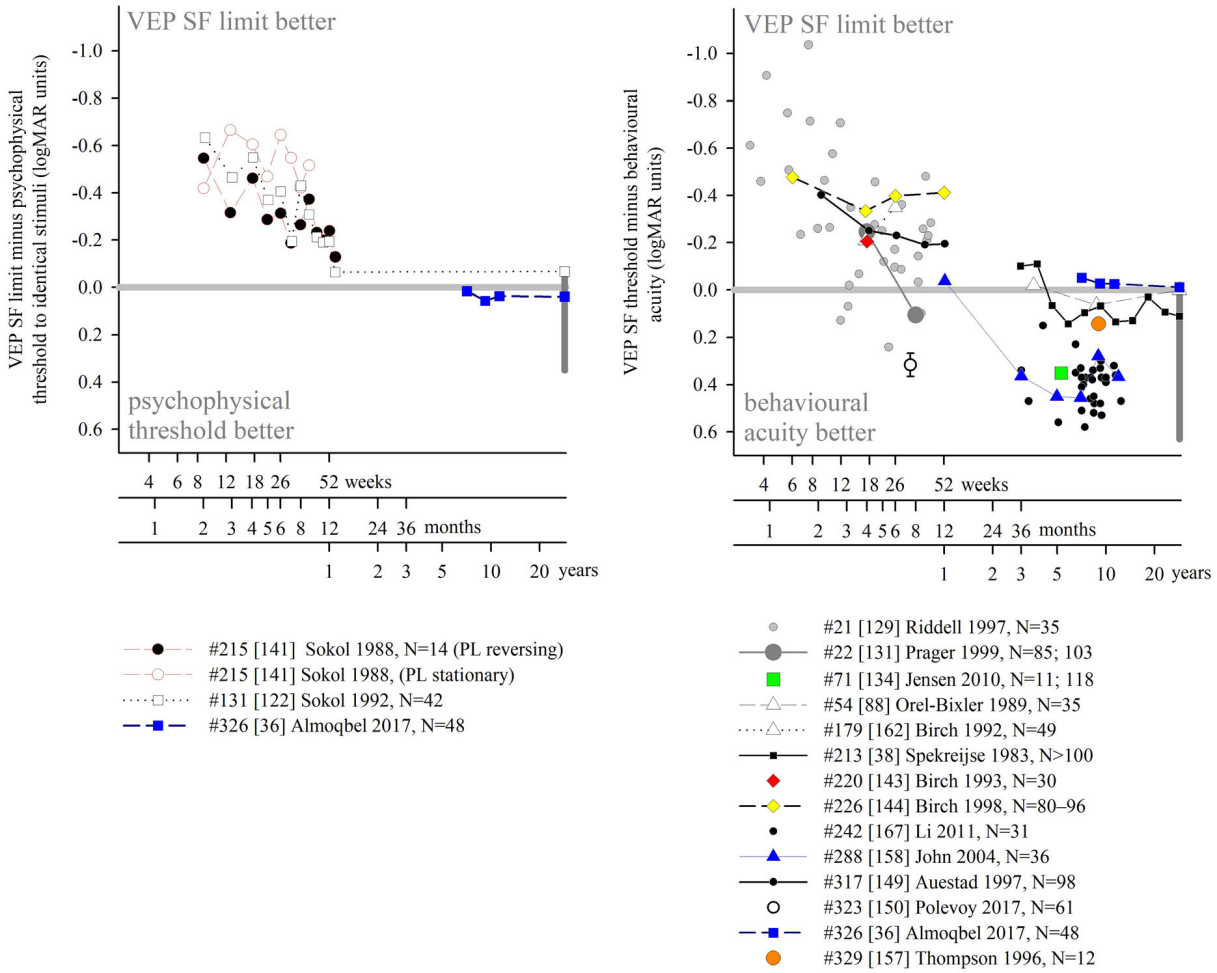
Illustration of difference between VEP SF limits and acuity and its change with age. Left panel: psychophysical acuity tests using identical stimuli to VEP test. Right panel: behavioural tests using age-appropriate clinical acuity tests. Solid grey horizontal line indicates no difference. Solid vertical line at adult ages indicates typical range of differences reported in healthy, normally sighted adults (see Fig. 5). Error bars represent SEM. PL: preferential-looking

Two groups have compared VEP SF limits with psychophysically measured thresholds to identical stimuli in infancy and childhood. Sokol et al. undertook a mixed cross-sectional and longitudinal study in 14 typically developing infants between 2 and 13 months. VEP SF limits improved from 4 to 19 cpd, while preferential-looking thresholds improved from 1 to 14 cpd over the same age range (Fig. 7, left). Some infants were also tested with a stationary version of the preferential-looking task, i.e. the stimulus did not reverse, which gave similar but slightly poorer thresholds [141]. The same group compared VEP SF limits and preferential-looking thresholds in 42 typically developing infants aged 2–13 months with very similar results, showing VEP SF limits much better than behavioural thresholds at the youngest ages tested (around 0.6 log units better at 2 months): this difference diminishes with age, approaching zero difference by the end of the first year of life [122] and closely matching the near-zero difference observed in adults [141]. Using a temporal, 2-alternative forced-choice staircase procedure, psychophysical thresholds were compared with VEP SF limits using identical stimuli, albeit with a smaller field size for the psychophysical measurements (4 × 4° vs. 10 × 10°) in 48 older children (6–12 years old) and adults. Both measures agreed closely, with slightly better psychophysical thresholds at all ages [36].

#### Non-identical stimuli—recognition acuity tests

We identified 14 studies which compared behavioural acuities with VEP SF limits in typically developing infants and children (Fig. 7 right). In all studies investigating children under 1 year, Teller acuity cards (TAC) or a digitally rendered similar test was used. At the youngest ages tested (≲10 weeks), VEP SF limits were consistently and markedly better, by 0.2–1.0 log units, than behavioural testing [129, 144, 149]. All used some form of extrapolation to define the VEP SF limit. This difference lessened with age: by 4 months of age, several studies found VEP SF limits to be better than behavioural thresholds by only around 0.2–0.35 log units [131, 143, 144, 149, 162]. Towards the end of the first year of life, the difference was less still but inconsistent, with some studies finding better behavioural acuity than VEP SF limits [131, 150] and some the opposite [129, 144, 149, 162].

Studies of children aged 3 up to adulthood found behavioural acuity either closely matched or up to 0.6 log units better than VEP SF limits [36, 38, 88, 134, 149, 157, 158, 167]. Three cross-sectional studies using a mixture of age-appropriate acuity tests found very closely matched thresholds, with a slight tendency for behavioural acuities to be better than VEP SF limits in older children and adults [36, 38, 88]. Four other studies found a more marked difference, with behavioural acuities better by around 0.4 log units [134, 149, 158, 167].

We were unable to find any extractable data comparing VEP SF limits with behavioural acuity for children aged 12–36 months, although a study of 35 typically developing infants and children aged 1–36 months noted VEP SF limits exceeded forced-choice preferential-looking acuity in the first year, but matched closely in the second and third years of life [157]. Only one cross-sectional study spanned these ages and found a marked change over this interval, with closely matched thresholds at 12 months, but markedly better behavioural thresholds by 36 months [158].

These data support the conclusion that VEP SF limits are much better than behavioural thresholds in the youngest, typically developing infants, but that this difference lessens with age, with no difference expected somewhere between the first and second year of life. From around 3–5 years, the same pattern is observed as is seen in healthy, normally sighted adults, i.e. behavioural acuity tends to be better than VEP SF limit. It was established earlier that normally sighted adults usually have behavioural acuity which is better than their VEP SF limits by 0 to 0.6 log units. The difference shows high inter-subject and between-studies variability at all ages and depends on both the VEP SF limit technique and the behavioural acuity test.

### Correspondence of VEP SF limits with behavioural thresholds in normally sighted adults with artificially degraded vision

**Fig. 8:**
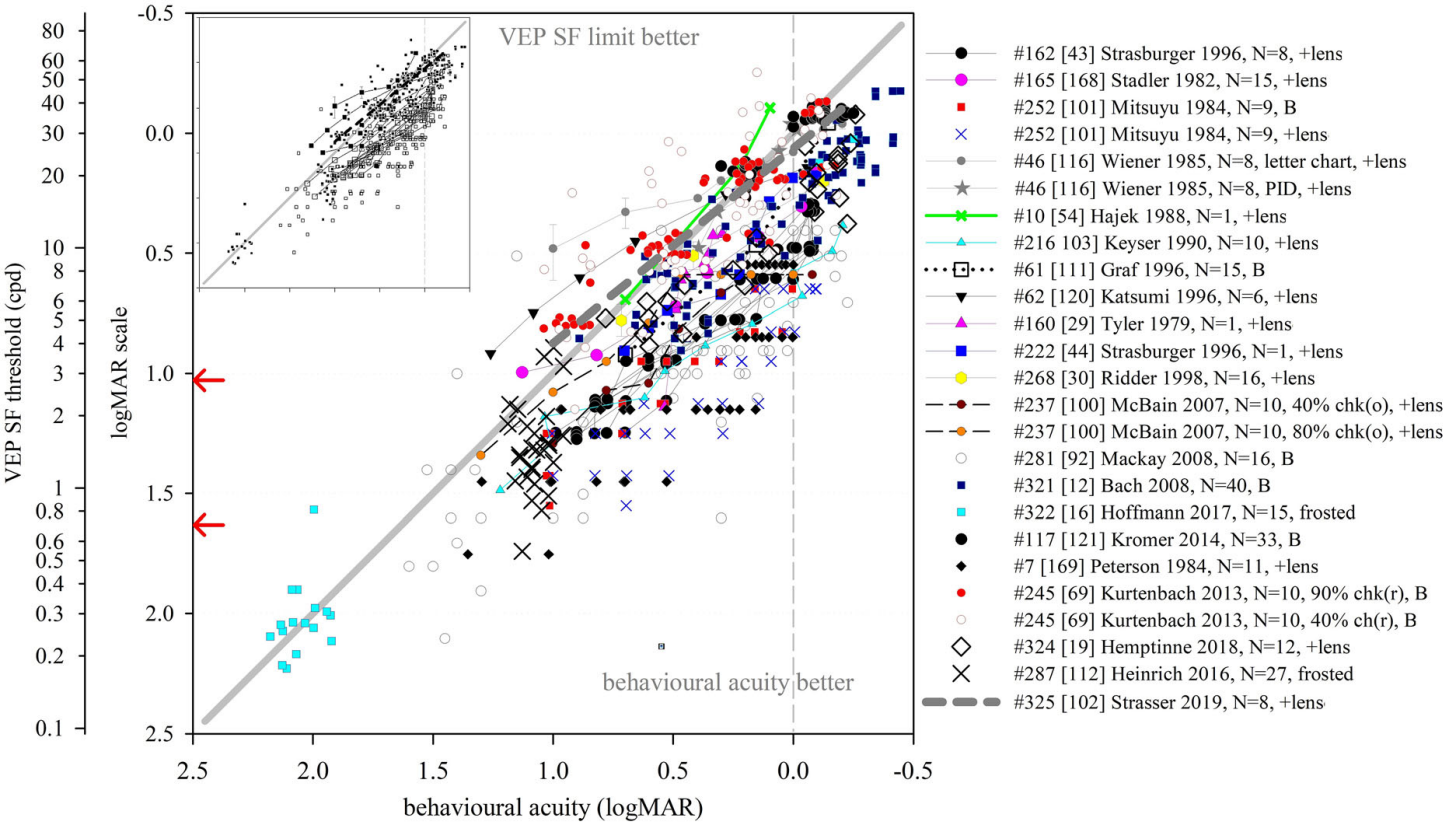
VEP SF limit versus behavioural acuity in healthy, normally sighted adults with artificially reduced acuity. Legend indicates study ID, number of subjects (*N*), and means of reducing acuity. Solid grey line: equality. Vertical dashed grey line: 0.0 logMAR. PID psychophysical test with stimuli identical to that used for VEP SF limit. Lines join symbols representing individual subjects if known, or join mean data from the same group of subjects. Where adjustments for behavioural versus VEP offsets were used [12, 16, 69, 112], these have been removed to allow comparison with unadjusted data from other studies. Red arrows at the SF axis indicate the two ISCEV standard checkwidths, 60′ and 15′ (0.71 and 2.8 cpd). Inset: the same data are presented to illustrate a closer VEP–behavioural match for those studies using extrapolation techniques (closed symbols) than those using the finest SF technique (open symbols)

We identified 21 studies where normally sighted adults had both behavioural acuity and VEP SF limits measured, while their vision was degraded using either Bangerter occluding foils [12, 69, 92, 101, 111, 121], plus lenses [19, 29, 30, 43, 44, 54, 100–103, 116, 120, 168, 169], scatter transparencies [16] or frosted panes [112]. Where possible, data were extracted and converted into logMAR units for both behavioural acuities and for VEP SF limits. If adjustments for behavioural versus VEP offsets had been applied [12, 16, 69, 112], these were removed to allow comparison with unadjusted data from other studies (Fig. 8).

Fifteen of the 21 studies found typical VEP SF limits to be poorer than perceptual acuity by around 0.2–0.6 log units [12, 19, 29, 30, 43, 44, 54, 92, 100, 101, 103, 111, 112, 168, 169]. Most of these studies also found this behavioural acuity versus VEP SF limit offset to be constant over the range of acuities assessed [12, 19, 43, 44, 54, 92, 101, 111, 169], albeit with a ceiling effect evident in one study which found the same VEP result for all acuities better than 0.5 logMAR (using 80% contrast checks), and similarly for acuities better than about 0.2 logMAR (for 40% contrast checks) [100]. This consistent behavioural acuity versus VEP SF limit offset was evident despite large differences in methods, i.e. VEP pattern, contrast, number of SFs, process of determining threshold, behavioural test and means of degrading vision. Four of these 15 studies found the behavioural acuity versus VEP SF limit offset changed over the range of acuities assessed, with the offset narrowing towards poorer acuities in three [30, 103, 168] but widening markedly in one study using a continuous sweep VEP technique to compare VEP SF limit with psychophysical acuity using an identical stimulus in one defocussed individual [29].

Two further studies found poorer behavioural acuities than VEP SF limits at all acuities assessed, but most marked at the poorest acuities measured [116, 120].

Four further studies found approximately equal behavioural acuities and VEP SF limits, i.e. an offset of approximately zero [16, 69, 102, 121]. One of these investigated subjects with markedly degraded acuity (≈ 2.0 logMAR) [16]; this close-to-zero offset was different from the 0.232 logMAR offset found using identical techniques in a large group of subjects degraded to acuities of ≈ 1.0 logMAR or better [12] and may therefore indicate a different calibration factor for the low vision range. Three further studies, all using high contrast patterns, also found little or no offset, with VEP SF limits even being slightly better than behavioural measures at the poorest acuities (≈ 1.0 logMAR or better [69], or at around 0.3 logMAR [121]). The third used transient on/offset VEPs fitted with a parabolic or modified Ricker function in eight defocused subjects to compare with Snellen chart acuities of 1.0 logMAR and better [102].

One study directly compared plus lenses with Bangerter foils and found Bangerter foils gave slightly better (by around 0.15 log units) VEP SF limits than plus lenses for the same behavioural acuities [101]. While not specifically identified in that study, optical defocus can cause spurious overestimation of both perceptual and VEP SF limits to periodic stimuli due to the emergence of a phase-inverted, lower contrast image as dioptric blur increases [170, 171].

One study incorporated two behavioural tests, a letter chart and psychophysical acuity using the same stimulus as for the VEP. VEP SF limits closely matched psychophysically measured acuity across all acuities assessed (≈ 0–0.4 logMAR), while VEP SF limits were markedly better than letter chart acuity at poor acuities, but somewhat worse at good acuities [116].

### VEP SF limits in clinical conditions

This section aims to compile what is known about VEP SF limits in a range of clinical conditions affecting vision such as cataract or macular disease, or in patients with clinical signs such as nystagmus, to establish its accuracy and precision and to comment on the suitability of using VEP SF limits to estimate acuity.

#### Heterogeneous patient groups

Several studies of VEP SF limits in patients investigated heterogeneous patient groups in order to establish real-life utility of the technique. General findings from such studies are presented initially: subsequent sections present findings by specific conditions or signs, ordered in an anterior to posterior direction along the visual pathway, i.e. beginning with conditions which affect optical input (media opacities, refractive error, nystagmus), then retinal and macular disease, followed by diseases of the optic nerve and finally covering conditions where the primary lesion is cerebral, i.e. amblyopia and neurological conditions. NOVL (non-organic vision loss) is treated last.

**Fig. 9:**
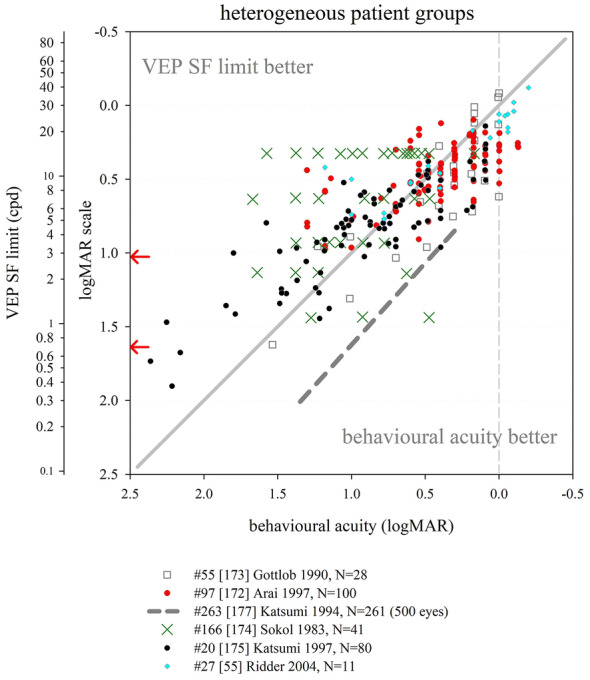
VEP SF limit versus behavioural acuity in heterogeneous groups of patients. Solid grey line: equality. Dashed vertical grey line: indicative normal behavioural acuity limit (0.0 logMAR). Red arrows at the SF axis indicate the two ISCEV standard checkwidths, 60′ and 15′ (0.71 and 2.8 cpd)

We identified eight studies of VEP SF limits in heterogeneous patient groups; data were exractable from six studies (Fig. 9). VEP SF limits were better than behavioural acuities in 329 ophthalmic patients (8–85 years) with diverse conditions impairing their vision; this overestimation was more pronounced at poorer acuity levels, e.g. VEP SF limits around 0.65 logMAR when behavioural acuity was around 1.5 logMAR [118]. Average VEP SF limits were similar to average behavioural acuities in 100 patients (7–90 years) with diverse conditions impairing their vision, but VEP SF limits exceeded acuity at poorer acuity levels, e.g. VEP SF limits around 0.7 logMAR when behavioural acuity was around 1.0 logMAR. The gap lessened as acuity improved; for patients with behavioural acuities better than about 0.45 logMAR, VEP SF limits fell short of behavioural acuities [172]. Similarly, VEP SF limits in 11 patients (3–81 years) with poor acuity due to diverse conditions were poorer by around 0.14 logMAR on average compared with letter chart acuity; however, for the poorest acuities (≈ 1.0 logMAR), VEP SF limits exceeded behavioural thresholds by around 0.7 logMAR: again, this gap lessened as acuity improved [55]. A sweep VEP method was highly successful (> 95%) and strongly correlated with behavioural acuity in a group of 135 patients aged 3 weeks to 11 years with diverse visual disorders. Behavioural acuities were typically around 0.1 logMAR better than VEP SF limits (Fig. 9), but the age span included ages when VEP–behavioural differences in typically developing children still reverse (cf. Fig. 7) [173]. VEP and a forced-choice preferential-looking technique had approximately equally success in children < 2 years old for binocular testing, but for monocular testing, the VEP technique (transient; six checkwidths with SF_f_ 5.7–240 cpd) was markedly more successful in children aged 3 years or under [174]. In the same study, for 41 patients (median age 1 year) with visual problems, binocular VEP SF limits were almost always better than behavioural acuities (average of 0.64 vs. 1.01 logMAR)—again, this age span typically shows a marked reversal in VEP–behavioural differences (cf. Fig. 7) [174]. Monocular VEP SF limits recorded from 80 paediatric patients (1.5 months to 12 years) with diverse visual disorders and a broad span of acuities agreed closely with behavioural acuities measured using a stationary version of the VEP stimulus and a forced-choice, preferential-looking or pointing/verbal responses staircase procedure (Fig. 9). As in the studies above, VEP–behavioural difference varied with acuity: for patients with the poorest behavioural acuities (≈ 2.25 logMAR), VEP SF limits were better (≈ 1.6 logMAR), while the opposite was found for patients with good acuity (≈ 0.1 logMAR), whose VEP SF limits were poorer at around 0.4 logMAR. Over 95% of patients had VEP and behavioural measures within ± 0.3 logMAR units [175]. Since the study group ages spanned those when VEP–behavioural differences typically reverse markedly (cf. Fig. 7), it is likely that some of the change in VEP–behavioural difference with acuity is due to the normal VEP–behavioural difference changing with age observed in healthy children [175].

The studies described above follow a pattern of VEP SF limits overestimating acuity at poor acuity but matching more closely as acuity improves. Two studies deviated from this pattern. One study of 42 children (4–116 months) with visual impairment due to multiple, diverse causes reported mean behavioural acuity better than mean VEP SF limit (0.89 vs. 1.16 logMAR) [176]; ages spanned those when VEP–behavioural differences typically reverse markedly (Fig. 7). Also, behavioural (forced-choice preferential-looking) tests were more successful than VEP SF limits extrapolated from transient (3.8 rps) VEPs (41/42 vs. 27/42), with particularly poor VEP success in those with nystagmus or seizure disorders. In the largest available study of patients, VEP SF limits were consistently poorer than behavioural acuity by about 0.6 logMAR units across all acuities assessed from 500 eyes of 261 patients (8–88 years): however, unlike other clinical studies, the threshold criterion was the finest SF to produce a reliable VEP, with the finest available checkwidth as large as 10′ (4.2 cpd, 0.85 logMAR) [177] (Fig. 9).

Despite an identical stimulation protocol and very similar patient groups, the two largest studies had markedly different findings: extrapolation to a 0 μV threshold [118] using a logarithmically scaled SF axis produced substantially better VEP SF limits than the finest SF technique [177].

In summary, data from adult and older paediatric patients show that VEP SF limits exceed behavioural acuity in patients with poor acuity. In some studies, this gap lessens as acuity improves, with the two measures matching at around 0.3–0.5 logMAR [55, 172]: for acuities better than this, VEP SF limits may underestimate behavioural acuity. Comparing Figs. 8 and 9 suggests that pathologies impairing visual acuity do not cause quite the same VEP SF limit–behavioural acuity relationship as that found for artificially blurred, healthy adults. General findings in younger paediatric patients are complicated by two factors: firstly, unlike adult patients, it cannot be assumed that the behavioural acuity measure is the gold standard. Secondly, the VEP–behavioural difference is known to alter markedly over the first 3 years of life in typically developing infants and children (cf Fig. 7). Studies presenting VEP–behavioural differences in ages which include both under- and over-3-year olds may therefore confound the impact of pathology with expected physiological development.

#### Opacities

**Fig. 10:**
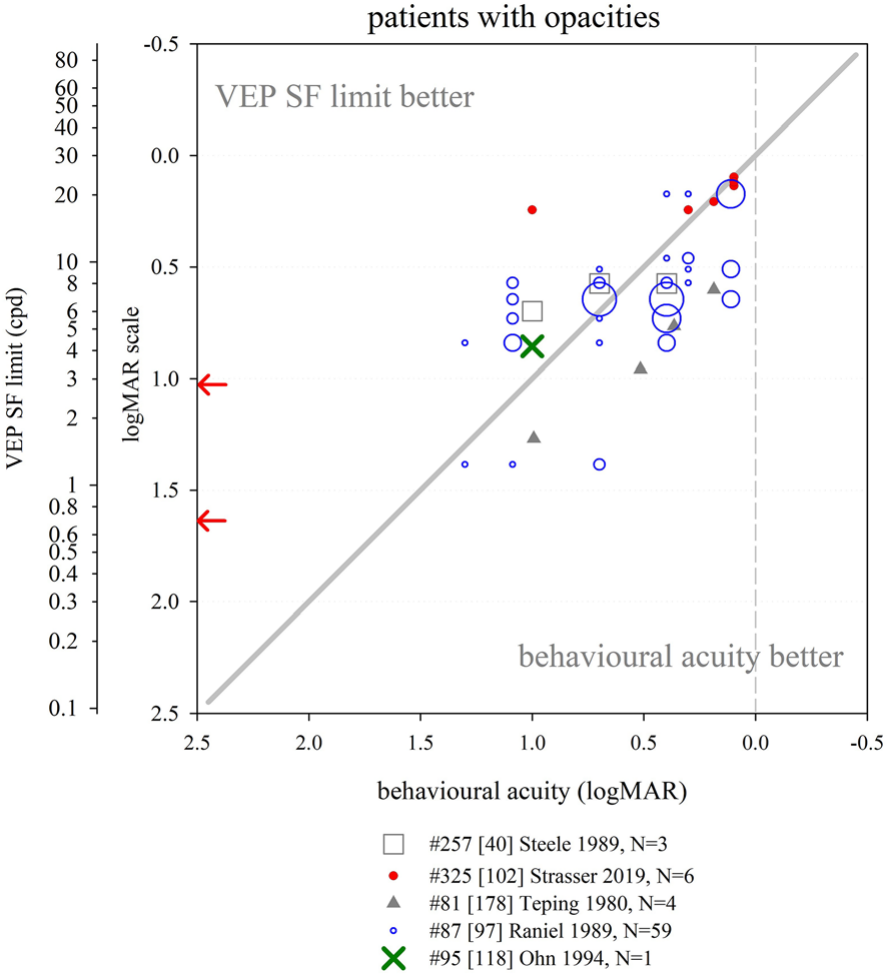
VEP SF limit versus behavioural acuity in patients with opacities. Open blue circle size reflects the number of subjects with any particular pair of results. Solid grey line: equality. Dashed vertical grey line: indicative normal behavioural acuity limit (0.0 logMAR). Red arrows at the SF axis indicate the two ISCEV standard checkwidths, 60′ and 15′ (0.71 and 2.8 cpd)

We identified six studies where VEP SF limits and behavioural acuities were measured in patients with media opacities such as cataract or vitreous opacities, five with extractable data (Fig. 10). A large study of 59 patients with varying degrees of cataract (*N* = 56) or vitreous haemorrhage (*N* = 3) using a finest SF criterion found average VEP SF limits only around 0.1 log unit poorer than behavioural acuities, but relatively wide 90% limits of agreement of around ± 0.5 logMAR [97]. In a group of 13 patients with cataracts, the average VEP SF limit was 0.26 log units better than behavioural acuities: one example patient (Fig. 10) had a VEP SF limit 0.144 log units better than their behavioural acuity of 1.000 logMAR [118]. Similarly, two smaller patient groups (*N* = 6; 5 cataract, 1 vitreous opacity [102]; *N* = 3; 2 cataract, 1 vitreous haemorrhages [40]) had generally close agreement between VEP SF limit and behavioural acuity. One study found VEP SF limits in four patients with cataracts consistently poorer than behavioural acuities by around 0.4 log units using a relatively dim (10 cd m^−2^), low-contrast (20%) stimulus [178]. A case series of six aphakic infants had transient VEP SF limits which correlated with single letter visual acuity [179].

Collectively, these data suggest that media opacities impair VEP SF limits and behavioural acuities similarly, and the trends seen in this small group of five studies are similar to those seen in healthy, normally sighted adults with artificially reduced acuity using plus lenses or Bangerter foils (Fig. 8). This supports the assertion that pathologies affecting the anterior part of the eye degrade the VEP stimulus akin to blurring [169].

#### Nystagmus

Pattern-reversal VEPs are degraded in patients with nystagmus, whereas pattern-onset VEPs are less affected [180, 181], probably because motion adaptation caused by nystagmus-induced retinal slip is lower for pattern onset than for pattern-reversal stimulation which has a higher duty-cycle of pattern-presence and hence retinal image motion [182]. It is therefore likely that VEP SF limits will be affected by the presence of nystagmus, and that the effect size will depend on the choice of on/offset or reversal, as well as orientation of gratings. However, we did not identify any studies which described changes in VEP SF limits with choice of on/offset or reversal, or with orientation of gratings, in patients with nystagmus. Twenty-six children with mild or moderate nystagmus in association with other diagnoses, part of a cohort of 175 children, had worse VEP “scores” (deficit in log units between an individual’s VEP SF limit and limits from age-expected norms; pattern reversal) by 0.77 log units than children without nystagmus (0.43 log units); similar deficits were found with behavioural testing (Teller Acuity Cards) with deficits of 0.86 and 0.52, respectively [183]. A large (*N* = 172) group of young (median age 1 year) patients with heterogeneous causes for vision loss had better VEP SF limits (pattern reversal) on average than behavioural acuity (0.33 vs. 1.01 logMAR, Fig. 9): in five patients with nystagmus, this was reversed and the VEP SF limits were about 0.15 logMAR units poorer than behavioural acuity [174]. Using horizontal gratings rather than checkerboards may improve success rates with VEP SF limits: having established VEP SF limits (checkerboard reversal) in only 28/42 (67%) paediatric patients with a broad range of aetiologies (22/42 including nystagmus) [176], the authors reported improved success in a subsequent study of 38 similar patients [184]. A sub-group (17/38) had nystagmus and so were tested with horizontal bar gratings (reversing) rather than reversing checkerboards, and VEP SF limits were established in 14/17 (82%) [184]. Mean VEP SF limits from fellow eyes were similar when six children exhibiting latent nystagmus were removed from the original group of 12, suggesting the choice of horizontal, reversing sinusoidal gratings were robust to nystagmoid blur [157].

Despite the well-established fact of more robust transient VEPs to on/offset than reversal stimuli, and the adoption of horizontally rather than vertically oriented gratings in clinical practice [185] for patients with horizontal nystagmus [186], there appears to be surprisingly little evidence of the effects of these stimuli changes on VEP SF limits in those with nystagmus.

#### Refractive error

**Fig. 11:**
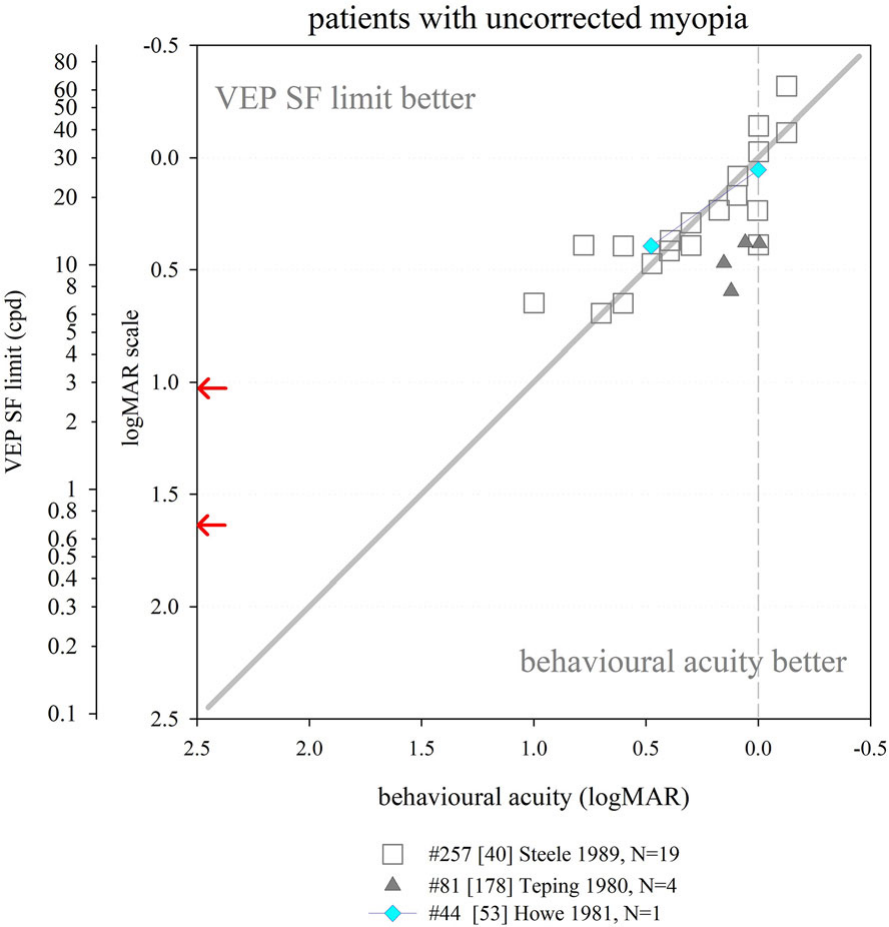
VEP SF limit versus behavioural acuity in patients with uncorrected myopia. Solid grey line: equality. Dashed vertical grey line: indicative normal behavioural acuity limit (0.0 logMAR). Red arrows at the SF axis indicate the two ISCEV standard checkwidths, 60′ and 15′ (0.71 and 2.8 cpd)

This section aims to identify the effect of uncorrected refractive errors on the VEP SF limit, and its relationship with behavioural acuity which is reasonably expected to be similar to effects of artificial blur in healthy adults (see above). Four studies were identified which measured VEP SF limits in uncorrected myopes; data were extractable from three of these. VEP SF limits recorded from 19 of 34 uncorrected myopic adults correlated well with behavioural acuities (Fig. 11): the 15 patients from whom no VEP SF limits were obtained (VEPs absent, or present only to the largest grating pattern (96′, 0.3 cpd, 1.98 logMAR)) had behavioural acuities from 1.2 to poorer than 1.6 logMAR [40]. For four uncorrected myopes, VEP SF limits were closely correlated and on average 0.37 log units poorer than behavioural acuity, but note the relatively dim (10 cd m^−2^), low-contrast (20%) VEP stimulus [178]. One adult myope had VEP SF limits of 27 and 12 cpd (0.054 and 0.394 logMAR) with and without refractive correction, respectively, and behavioural acuities of 0.000 and 0.477 [53]. Seemingly similar findings to those above are described for five patients with (corrected) high myopia and no other disorder, with VEP SF limits underestimating behavioural acuity: in four of the five patients, this difference was less than 0.3 log units [172].

Relatively few data were found describing the effect of refractive error on VEP SF limits, and over a more limited acuity range (normal to around 1.0 logMAR) compared to the “inverse” situation described in Fig. 8, where adults with no or little refractive error are blurred with plus lenses. As far as the two can be compared, they seem to indicate a similar relationship and highlight the importance of accurate refraction for measuring VEP SF limits.

#### Retinal conditions

**Fig. 12:**
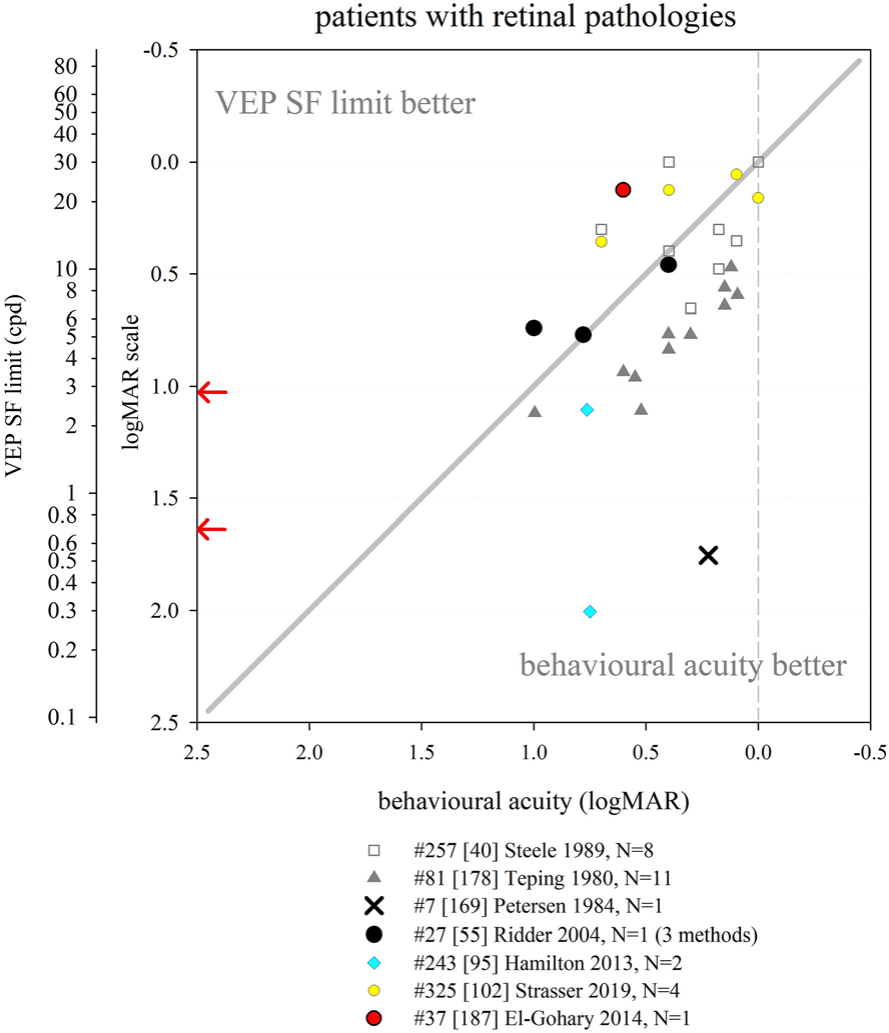
VEP SF limit versus behavioural acuity in patients with various retinal pathologies. Solid grey line: equality. Dashed vertical grey line: indicative normal behavioural acuity limit (0.0 logMAR). Red arrows at the SF axis indicate the two ISCEV standard checkwidths, 60′ and 15′ (0.71 and 2.8 cpd)

We identified seven studies with data comparing VEP SF limit with behavioural acuity in patients with primarily or solely retinal dysfunction not restricted to the macula (Fig. 12). Eight patients (five with diabetic retinopathy, one with juvenile X-linked retinoschisis, one with central serous retinopathy and one with retinitis pigmentosa) had widely varying differences between VEP SF limits and behavioural acuities, including two with no difference [40]. This pattern of inconsistent agreement was also found for nine further patients, four with retinitis pigmentosa [102], one with diabetic retinopathy [55], two with a rod-cone dystrophy [55, 95] and one with a cone abnormality [187]. In a study of 11 patients with either retinitis pigmentosa or choroidal atrophy, behavioural acuity was systematically better than VEP SF limit: however, this study employed a relatively dim (10 cd m^−2^) and low-contrast (20%) VEP stimulus [178]. One further patient, used to illustrate a large discrepancy between VEP SF limit and behavioural acuity associated with low amplitude VEPs, had mild acuity loss (0.22 logMAR) but a very poor VEP SF limit (1.75 logMAR) [169]; a similar discrepancy was also reported in a 6-year-old child with a cone dystrophy [95]. In contrast, in 14 patients with retinal dystrophies, the average VEP SF limit was 0.126 log units better than behavioural acuity [118]. These findings suggest that—similar to patients with opacities—retinal disease impairs VEP SF limits and behavioural acuities similarly, albeit with wider disparities. The trends seen in these studies of patients with retinal disease are similar to those in healthy, normally sighted adults with artificially reduced acuity using plus lenses or Bangerter foils (Fig. 8). While a retinal pathology may degrade portions of the stimulus, the spatial integration which takes place along the visual pathway may minimise the evidence of localised retinal dysfunction on the VEP. Conversely, preservation of even a small (e.g. 2 degrees) central portion of the fovea may still afford good or even excellent behavioural acuity, but markedly reduce or even eliminate the VEP [169]. A simulation of this effect has been described [29, Fig. 3B].

#### Macular conditions

**Fig. 13:**
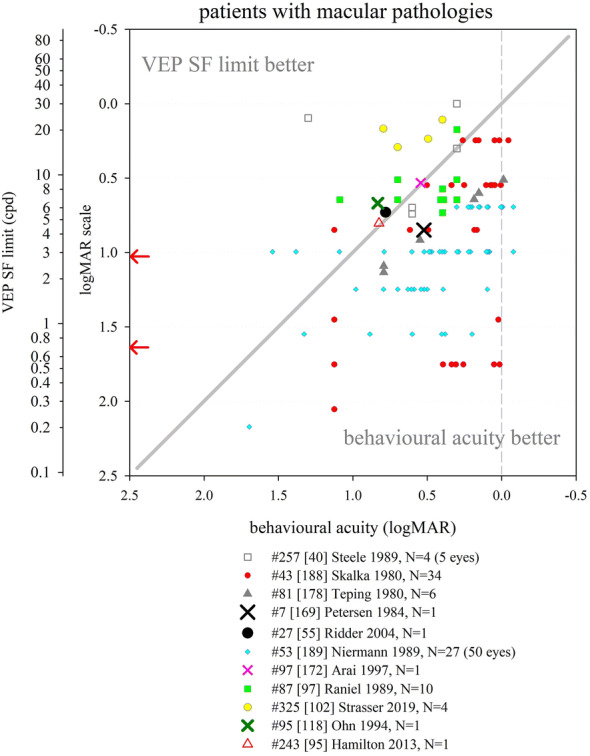
VEP SF limit versus behavioural acuity in patients with macular pathologies. Solid grey line: equality. Dashed vertical grey line: indicative normal behavioural acuity limit (0.0 logMAR). Red arrows at the SF axis indicate the two ISCEV standard checkwidths, 60′ and 15′ (0.71 and 2.8 cpd)

We identified 11 studies which compared VEP SF limits with behavioural acuity in patients with macular disease (Fig. 13). Two studies with fairly large groups of patients found a preponderance of cases with VEP SF limits poorer than behavioural acuities: 32/34 patients [188] and 47/50 eyes of 27 patients [189], respectively. Subjects were adult or older children, so behavioural acuity could be considered as gold standard. A smaller study also found poorer VEP SF limits than behavioural acuity in six patients with macular disease; however, this could at least partly be due to the relatively dim (10 cd m^−2^), low-contrast (20%) VEP stimulus [178].

Five studies presented individual patients with macular disease with quite closely matched VEP SF limits and behavioural acuities [55, 95, 118, 169, 172], one of which also noted that 28/35 eyes (80%) with macular diseases had a visual acuity difference between the two acuities within 1.0 octave (0.3 log units) (data for the 35 eyes not extractable) [172]. In a large group of 67 patients with macular disease, the average VEP SF limit was only 0.04 log units poorer than behavioural acuities, but showed wide variability: one example patient had a VEP SF limit 0.163 log units better than behavioural acuity [118]. Additional small groups of patients (*N* = 4 and 10, respectively) with macular pathology were noted to have approximately equal VEP SF limits and behavioural acuities [40, 97]. One study found VEP SF limits better than behavioural acuity in all four patients studied [102].

Macular disease appears predominantly to result in VEP SF limits which are substantially poorer then behavioural acuity, but findings are sufficiently scattered to suggest that any correlation is very weak (Fig. 13). The extent of macula affected may govern the quality of this correlation: where disease affects only the fovea, the rest of the macula may continue to generate a VEP. Experiments with mimicked central scotomas showed a two degree scotoma only slightly affected the VEP SF limit, but VEP SF limits worsened as the scotoma increased in size: VEP amplitude was reduced at all SFs, reducing the slope of the linear amplitude extrapolation [29].

#### Optic nerve

**Fig. 14:**
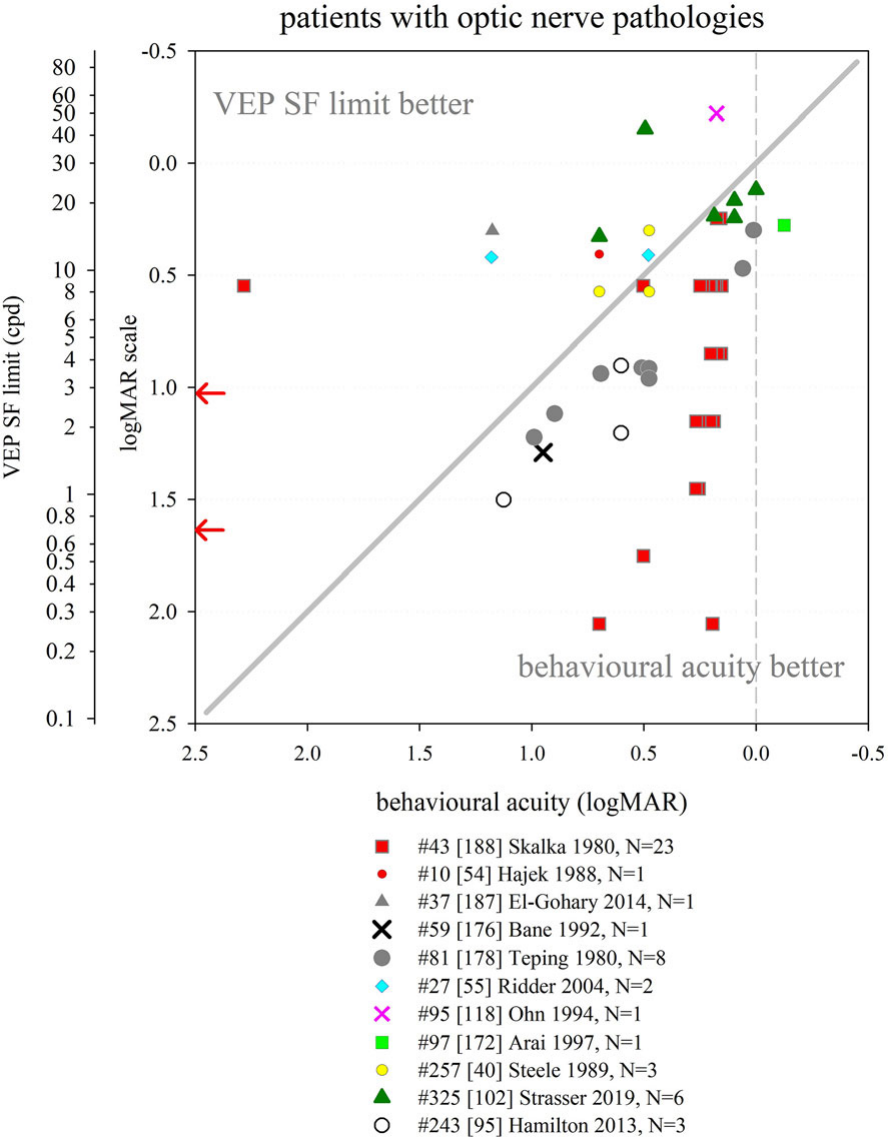
VEP SF limit versus behavioural acuity in patients with optic nerve pathologies. Solid grey line: equality. Dashed vertical grey line: indicative normal behavioural acuity limit (0.0 logMAR). Red arrows at the SF axis indicate the two ISCEV standard checkwidths, 60′ and 15′ (0.71 and 2.8 cpd)

We identified 11 studies with extractable data where patients with optic nerve disorders had both VEP SF limits and behavioural acuity compared (Fig. 14). In general, VEP SF limits were poorer than behavioural acuity. In a large group of patients (*N* = 68) with optic nerve disease, an average deficit of around 0.2 log units was noted in the VEP SF limits, but with wide discrepancies: this is illustrated with an example (Fig. 14) which did not follow the group trend, where a patient with behavioural acuity of 0.176 logMAR had an excellent VEP SF limit of − 0.22 logMAR [118]. VEP SF limits poorer than behavioural acuity was found in 22/23 eyes in a group of patients with a variety of optic nerve diseases including retrobulbar neuritis, ischaemic optic neuropathy, traumatic neuropathies and optic nerve tumours [188]; eight patients (four with optic atrophy, four with optic neuritis) had VEP SF limits poorer than behavioural acuity, albeit using a relatively dim (10 cd m^−2^), low-contrast (20%) VEP stimulus [178]. Specific examples where VEP SF limits were markedly poorer than behavioural acuity include a child with optic nerve disease but without nystagmus or seizure disorder [176] and a 47-year-old man with optic neuritis and excellent behavioural acuity (− 0.125 logMAR vs. VEP SF limit of 0.278) [172]. The latter study investigated a group of 27 patients with optic neuritis or optic atrophy and found poor correlation of behavioural acuity and VEP SF limit: only 17/27 had a difference within 1.0 octave (0.3 log units) [172]. Mean VEP “scores” (log reduction relative to age-expected norms) of 0.95 log units were worse than behavioural acuity (Teller Acuity cards) “scores” of 0.86 log units in children whose visual impairment included optic nerve atrophy [183].

Examples of closer matching of VEP SF limits and behavioural acuity, or of VEP SF limits better than behavioural acuity were noted in six patients with optic neuritis [102], in three patients, one with optic nerve hypoplasia, one with optic neuritis, and one with toxic optic neuropathy [40], in a patient with optic neuropathy [54], in two patients with optic nerve hypoplasia [55] and in a young (2.5-year old) patient with optic atrophy [187].

Optic nerve disease therefore seems to result in VEP SF limits which are usually, but not always, poorer than behavioural acuity, with evidence of wide scatter (Fig. 14). This may be related to the well-established phenomenon of optic nerve disease often reducing VEP amplitude and therefore SNR. Small-but-extant VEPs close to the SF limit are therefore likely to be mis-categorised as absent, worsening VEP SF limits, a situation which could be improved by employing longer recording times close to the SF limit to enhance SNR: however, most procedures employ fixed recording duration for every pattern size. For extrapolated VEP SF limits, lower amplitudes also lead to flattened spatial tuning functions [118, 172] and hence reduced slope of the linear amplitude extrapolation, increasing the error associated with the crossing point, especially if SF sampling is sparse towards the limit and/or if the SF axis is logarithmically scaled. This might explain the wide discrepancies between VEP SF limit and behavioural acuities noted in optic nerve disease [118, 172, 173, 177].

#### Amblyopia

**Fig. 15:**
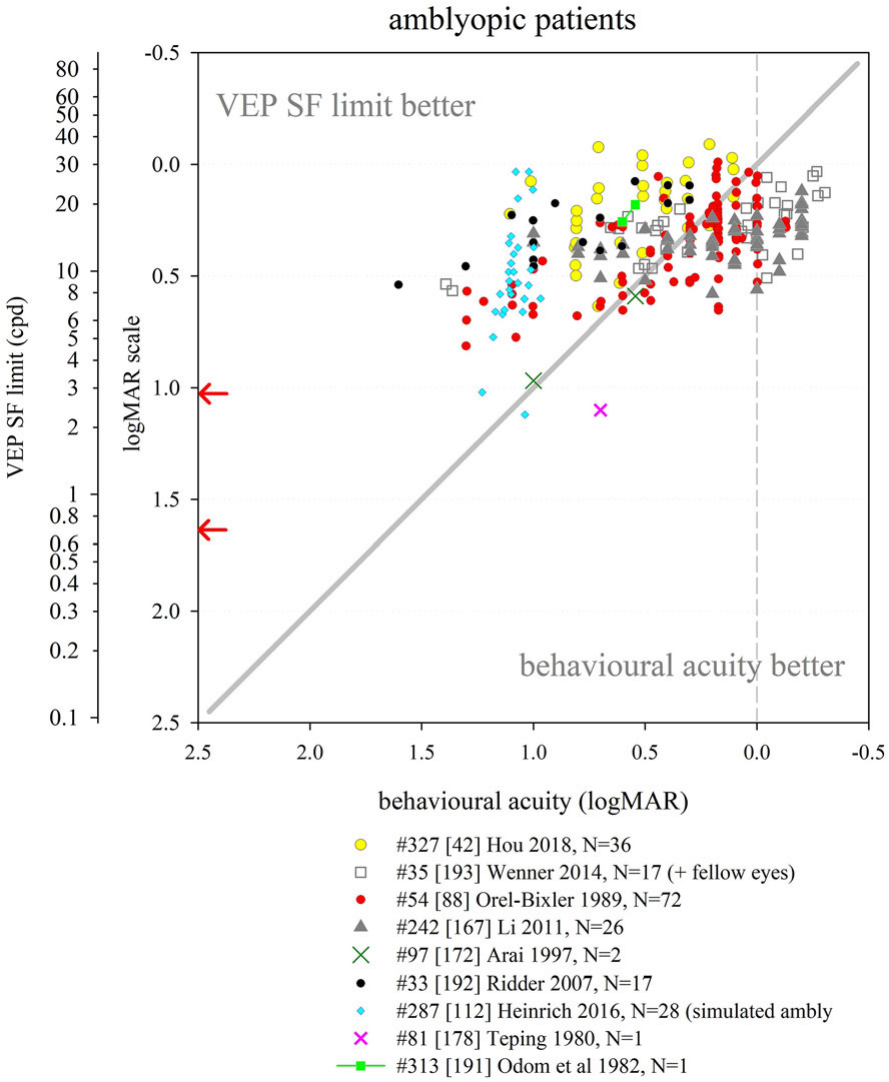
VEP SF limit versus behavioural acuity in amblyopic patients, some also with fellow eye data. Solid grey line: equality. Dashed grey line: indicative normal behavioural acuity limit (0.0 logMAR). Red arrows at the SF axis indicate the two ISCEV standard checkwidths, 60′ and 15′ (0.71 and 2.8 cpd)

Amblyopia—reduced optotype acuity measured from one or both eyes not exclusively attributed to a structural abnormality of the eye and due to impaired development of a normal cortical visual pathway—has been extensively investigated with VEPs because of the cortical pathway involvement and the need for objective tests at the typical ages of patients. We identified nine studies which measured VEP SF limits in children or adults with amblyopia (Fig. 15). Patients included adults and children. Some had a mixture of treated (patching/surgical) and untreated patients, but most studies did not state whether patients had ever been treated. VEP SF limits demonstrated rapid acuity improvements in two young (< 2 years) children due to patching of the better eye, but not in two older children: contemporary behavioural acuities were not measured [190]. Where both VEP SF limits and behavioural acuities were recorded, VEP SF limits were almost always better. VEP SF limits improved from 17 to 20 cpd, i.e. 0.257 to 0.180 logMAR 1 month before and after extraction of a subcapsular cataract and fitting of a soft contact lens aphakic correction, while behavioural acuities improved from 0.602 to 0.544 logMAR [191]. A detailed study of 72 amblyopic patients (2–61 years) with behavioural acuities (Bailey–Lovie chart) ranging from 0.4 to 1.6 logMAR in their amblyopic eye found VEP SF limits were better than optotype acuities: this difference became more marked as acuity worsened, with the VEP SF limit increasingly overestimating optotype acuity [88]. Similarly, VEP SF limits were around 0.5 logMAR units better than behavioural acuities of the poorer eye (0.3–1.6 logMAR) of 17 pre-treatment amblyopic patients. After 3–20 months of treatment, the 17 patients’ behavioural acuities matched their pre-treatment VEP SF limits (5–95th percentiles of difference − 0.24 to 0.15 logMAR). The authors concluded that VEP SF limits are a good predictor of post-therapy Snellen acuity [192]. Slightly better VEP SF limits than behavioural thresholds were found for the poorer eye of 26 amblyopic children aged 3 to 12 years: VEP SF limits exceeded behavioural acuities by about 0.1 log units across all acuities, but by much more at poorer acuity [167]. VEP SF limits were also found to be better (by 0.2 logMAR units) overall than behavioural (Landolt C) acuity in 17 adult amblyopic patients, and again, the difference was strongly dependent on underlying acuity, with the disagreement larger for poorer acuity [193]. Similarly, while VEP SF limits correlated with behavioural (Bailey–Lovie letter chart) acuity in 26 adult amblyopes, VEP SF limit was almost always better, an effect which was more marked for poorer acuity [42]. One study described two amblyopic patients with behavioural acuities (EDTRS chart, 0.544 and 1.000 logMAR) closely matched to their VEP SF limits (0.591 and 0.968 logMAR) [172], and only one study described a poorer VEP SF limit (1.1 logMAR) than behavioural acuity (0.70 logMAR) in an amblyopic adult, using a relatively dim (10 cd m^−2^), low-contrast (20%) VEP stimulus [178].

The evidence from these studies suggest that VEP SF limits are relatively insensitive to the acuity reduction seen in amblyopia when using an optotype-based acuity test, overestimating behavioural acuity markedly for poor optotype acuity, matching acuity at around 0.3–0.5 logMAR, and often underestimating acuity for acuities better than around 0.3 logMAR, as for normally sighted individuals. A similar mismatch has been extensively described for psychophysically measured grating acuity in amblyopia, while measures of Vernier acuity, either VEP-based or psychophysical, match optotype acuity more closely [194, 195]. A VEP SF limit represents a task-free threshold to high-contrast, repetitive stimuli and is therefore relatively robust to the higher neural noise and eccentric or unsteady horizontal fixation found in amblyopic vision [55]. VEP grating or checkerboard stimuli are also probably more robust to the “phase-scrambling” effect of amblyopia than are optotypes: a study of 27 adults whose vision was degraded to emulate the distorted and fragmented nature of amblyopic vision found VEP SF limits markedly exceeded behavioural thresholds by around 0.58 logMAR units, while no such overestimation was found for VEP SF limits from vision degraded with simpler blurring with frosted panes [112]. In conclusion, VEP SF limits are not optimal for monitoring amblyopia-associated visual acuity losses since they underestimate optotype-based acuity and are relatively insensitive to optotype-based acuity changes.

#### Neurological or structural brain abnormalities

VEP SF limits are used to infer acuity when neurological or structural brain abnormalities preclude behavioural testing, for example due to speech impairment, inability to point, poor or absent head or trunk control or eye movement disabilities such as gaze apraxia impairing fixation. Patients with these impairments may be mislabelled as visually impaired, and in some cases, VEP assessment may reveal otherwise hidden visual pathway capability. Patients with brain tumours, hydrocephalus, lissencephaly, microcephaly, delayed visual maturation (DVM), cerebral palsy (CP), periventricular leukomalacia (PVL), prematurity sequelae, seizures, or any of neurological disorders such as West syndrome, Aicardi’s Syndrome or neuronal ceroid lipofuscinoses may fall into this category.

For the populations in whom relative success has been reported, success rates of establishing VEP SF limits are generally higher than for behavioural methods. Success rates were 56/59 (95%) versus 41/59 (69%) in a group of patients with multiple, diverse neurological and visual disorders, aged 3–33 years; the 15 patients who could not undertake the preferential-looking grating acuity card test had a wide range of VEP SF limits (0.75–11 cpd, i.e. 1.6–0.44 logMAR), suggesting that the level of vision alone did not predict which test type would be possible [65]. Similarly, 167/173 (97%) children with CP, aged 6–48 months, provided a VEP SF limit while only 148/173 (85%) completed Teller Acuity Card testing, even though the behavioural test was undertaken first [196]. Only 54/76 (71%) children with CP, aged 2–19 years, could be reliably tested with optotypes; children with the most severe motor impairment were most unsuited to optotype testing due to upper motor limb dysfunction and/or speech impairment. VEP SF limits were established in 13 of the 22 (59%) children who could not undertake optotype testing, and failure to record a VEP SF limit was at least partly due to the trunk, head and neck instabilities, nystagmus and gaze apraxias or palsies prevalent in more severe CP which impair the child’s ability to maintain steady fixation on the VEP stimulus screen [197]. Success rates may be poorer when transient rather than steady-state VEPs are used, probably due to the much longer recording times required to find reproducible waveforms at slow reversal rates: 62/75 (83%) children (5–192 months) with multiple, diverse neurological impairments completed VEP SF limit testing, while 57/75 (76%) completed behavioural acuity testing [198]. However, VEP SF limits based on transient VEPs were established in 10/11 girls with Rett syndrome, none of whom could complete behavioural testing [199].

VEP SF limits established in 13/22 (59%) children with CP aged 2–19 years and who could not undertake optotype acuity testing showed a trend of worsening limits with increasing CP:0.30 logMAR to 0.45 logMAR for level 1 to level 5 (of motor impairment), although non-recordable VEP SF limits could be due to motor dysfunction (trunk, head and neck instabilities, nystagmus, gaze apraxias or palsies) rather than visual impairment [197]. Poorer-than-normal VEP SF limits (“normal” defined by 50 healthy, age-matched subjects) were found in 26 of 37 (70%) children aged 6–48 months with CP but without ophthalmological complaints and with normal fundi, and were more common in children with more severe motor impairment [200], as described elsewhere [197]. Poorer-than-normal VEP SF limits were also found in 29/30 children (6–108 months) with West syndrome [201].

**Fig. 16:**
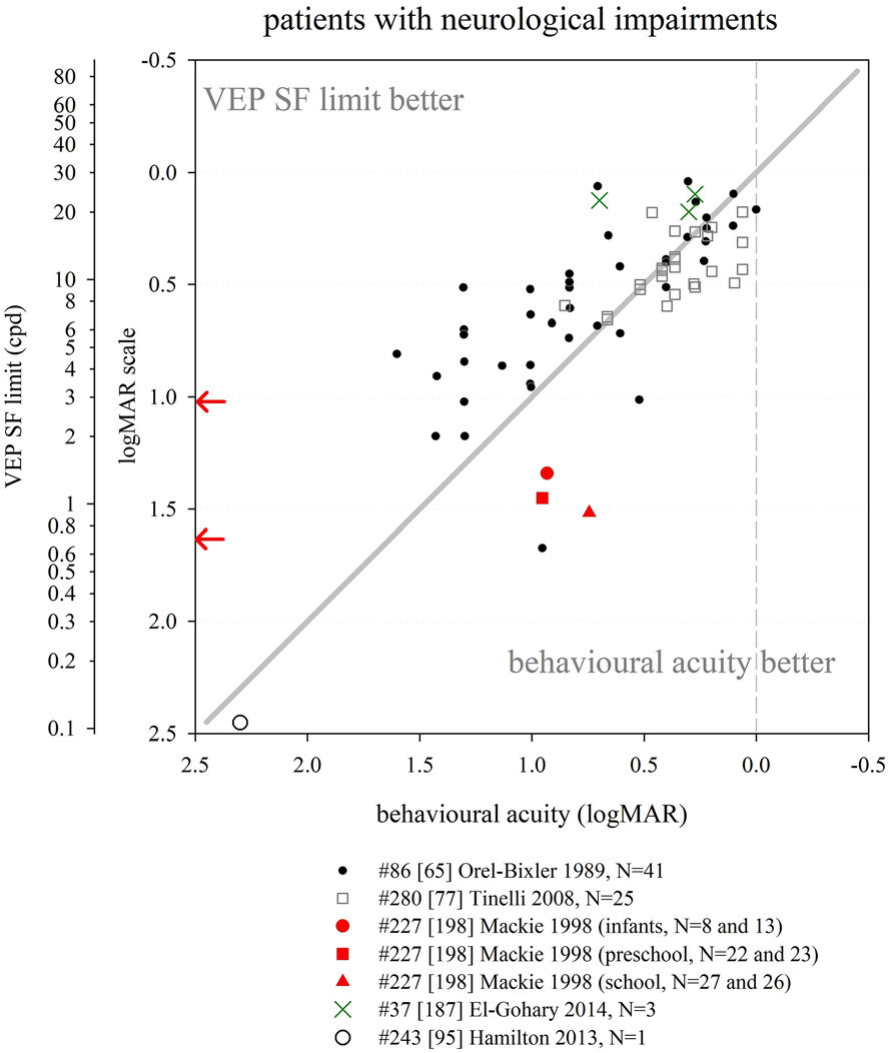
VEP SF limit versus behavioural acuity in patients with neurological impairments. Solid grey line: equality. Dashed grey line: indicative normal behavioural acuity limit (0.0 logMAR). Red arrows at the SF axis indicate the two ISCEV standard checkwidths, 60′ and 15′ (0.71 and 2.8 cpd)

Where comparisons with behavioural tests were made, reasonable agreement with wide scatter was observed. If a trend was apparent, this generally indicated VEP SF limits better than behavioural acuity at poor acuity levels, with the two measures reaching closer agreement for patients with better acuity (Fig. 16). In 41 patients with multiple, diverse neurological and visual disorders, aged 3–33 years, VEP SF limits slightly exceeded behavioural acuity (forced-choice, preferential-looking grating acuity card test) at the poorest acuities tested, e.g. VEP SF limit of around 1.0 logMAR for behavioural acuity of around 1.5 logMAR. This mismatch lessened as acuity improved [65]. VEP SF limits fell within normal limits for around 40/167 children with CP, aged 6–48 months, with normal fundi and no ocular disease, and showed an improving trend with age. VEP SF limits were 0.208 logMAR better on average than behavioural thresholds (Teller Acuity Cards; preferential-looking, two-alternative, forced-choice, staircase procedure); the VEP–behavioural difference showed no correlation with age. Both VEP SF limits and behavioural acuities were poorer for children with more severe motor impairment: the VEP–behavioural difference was also greater for children with more severe motor impairment. Limits of agreement (5th–95th percentiles) were ± 0.35 logMAR (± 1.2 octaves) [196]. Very similar VEP SF limits and behavioural acuities (Teller Acuity Cards) were measured in a group of 29 children (9 months to 13 years) with PVL and CP, many of whom were born prematurely and had cognitive impairment: all but one had visual abnormalities. The VEP–behavioural difference tended to increase with age, with little difference on average for children under 1 year, but a tendency towards poorer VEP SF limits than behavioural acuity in older children. Limits of agreement (5th–95th percentiles) for the whole group were ± 0.27 logMAR (± 0.9 octaves) [77]. El-Gohary et al. [187] found three patients with CP to have better VEP SF limits than behavioural acuities by around 0.1, 0.2 and 0.6 logMAR units. A 9-year-old child with a craniopharyngioma had equally poor behavioural acuity and VEP SF limit [95].

Poorer VEP SF limits using transient on/offset VEPs without extrapolation (0.78–2.68 logMAR) than behavioural acuities (0.07–2.08 logMAR) were found in a group of 75 children (5–192 months) with multiple, diverse neurological impairments. VEP SF limits did not vary with age, but were poorer in children with a cortical site for the major visual pathway lesion than those with optical, retinal or optic nerve lesion sites. Both acuity measures were poorer in children with more severe motor or intellectual impairment [198].

Reasonable agreement between VEP SF limits and behavioural acuity, expressed as deviation from age-typical values, was found for a large group of paediatric patients with mixed ocular and neurological impairments; 48% of thresholds agreed within one octave (0.3 logMAR). Children with CP (*N* = 54) had poorer-than-normal VEP SF limits by 0.71 logMAR units on average, while behavioural thresholds were 1.01 logMAR units poorer-than-normal. Children with developmental delay (*N* = 75) had poorer-than-normal VEP SF limits by 0.57 logMAR units on average, while behavioural thresholds were 0.72 logMAR units poorer. Larger deviations from agreement were “contributed to” by the presence of developmental delay, CP or seizures [183].

VEP SF limits for transient VEPs and behavioural acuity were qualitatively described in 100 paediatric patients (3 months–8 years) with predominantly neurological impairments: 69/89 were “in agreement”, 14/89 (with predominately primary ocular abnormalities) had VEP SF limits which fell short of their behavioural acuity and 6/89 had VEP SF limits which exceeded their behavioural acuity [202].

##### Cerebral visual impairment (CVI)

CVI, recently defined as “a verifiable visual dysfunction which cannot be attributed to disorders of the anterior visual pathways or any potentially co-occurring ocular impairment” [203], can be misdiagnosed as a disorder that is behavioural or psychological in nature. VEP SF limits in patients with CVI have received specific interest and so is discussed separately from more general neurological or structural brain abnormalities. Definitions of CVI in the studies listed below all adhere to the general principle of bilateral acuity loss due to brain lesions, with normal ocular structures and pupillary reactions.

Studies which compared VEP SF limits with age-matched control children’s VEP SF limits found deficits in all [74, 79, 204] or most [205] of the patients investigated. Test–retest on a subset of 23 patients showed high reliability (*r*^2^ = 0.662, *P *= 0.0003) [204]; slightly better VEP SF limits were noted in CVI children using dim (20 cd m^−2^) versus bright (109 cd m^−2^) stimuli (10 cpd vs. 7.3 cpd), an effect not found in control children [126].

**Fig. 17:**
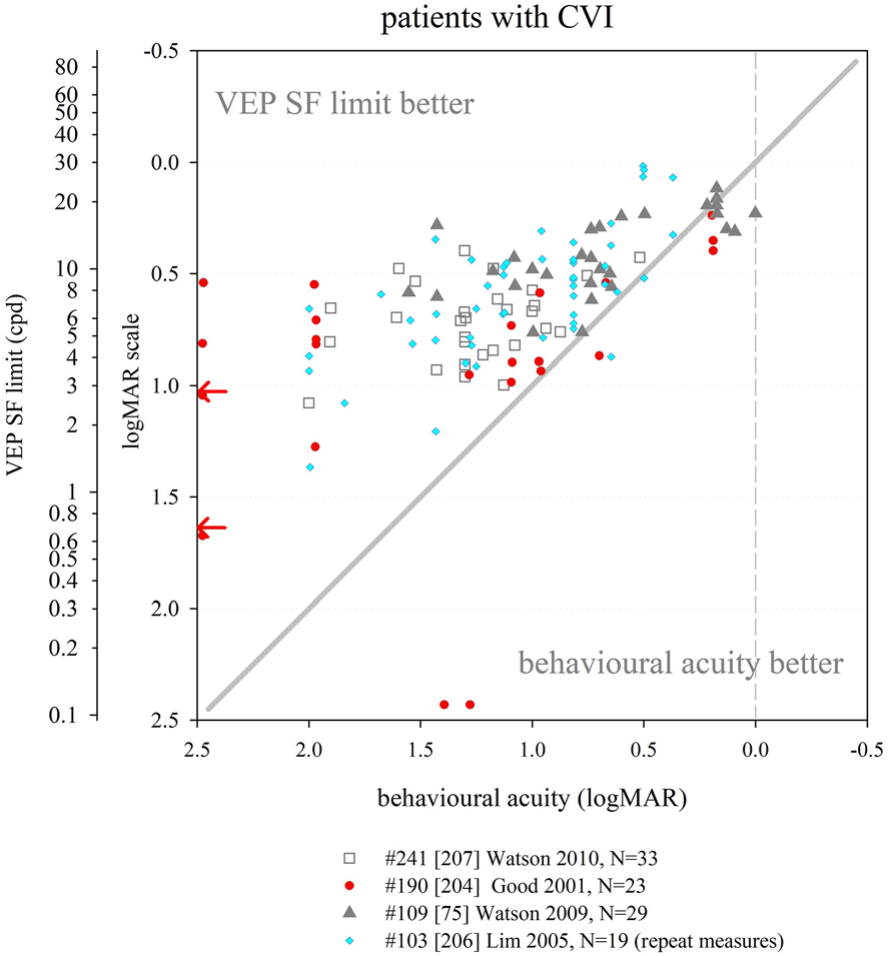
VEP SF limit versus behavioural acuity in patients with cerebral visual impairment (CVI). Solid grey line: equality. Dashed grey line: indicative normal behavioural acuity limit (0.0 logMAR). Red arrows at the SF axis indicate the two ISCEV standard checkwidths, 60′ and 15′ (0.71 and 2.8 cpd)

We identified four studies where both VEP SF limits and behavioural acuities were recorded from cohorts of CVI patients (Fig. 17). In all four, VEP SF limits and behavioural acuities were related, albeit with variable levels of agreement. VEP SF limits were generally better than behavioural thresholds, especially at poor acuity levels [75, 204, 206, 207].

Typically, both VEP SF limits and behavioural acuity (preferential-looking) acuity showed equal improvements in CVI patients during the developmental course [206]. It was noted that VEP SF limits are most useful in children with CVI who are difficult to engage or who make no or only fleeting eye contact; the authors also note that VEP SF limits can be astonishingly good for the child’s level of visually guided behaviour [206]. VEP SF limits matched closely to behavioural acuity measured 2–13 years later, suggesting a role for “predicting” developed acuity. However, this presumably simply reflects the difference in maturational curves of the two tests, and high variability might be misleading in some instances [207].

#### Non-organic visual loss

Non-organic visual loss (NOVL) refers to reduced visual function (here, specifically acuity loss) not caused by any organic lesion. Functional visual loss or medically unexplained visual loss are alternative terms. All three terms avoid assumptions about secondary gain or aetiology, which cannot be established via ophthalmic examination. Patients presenting with NOVL may or may not have voluntary control over their symptoms as they present along a spectrum from malingering/factitious disorders to somatisation/conversion (previously “hysterical”) disorders [208].

**Fig. 18:**
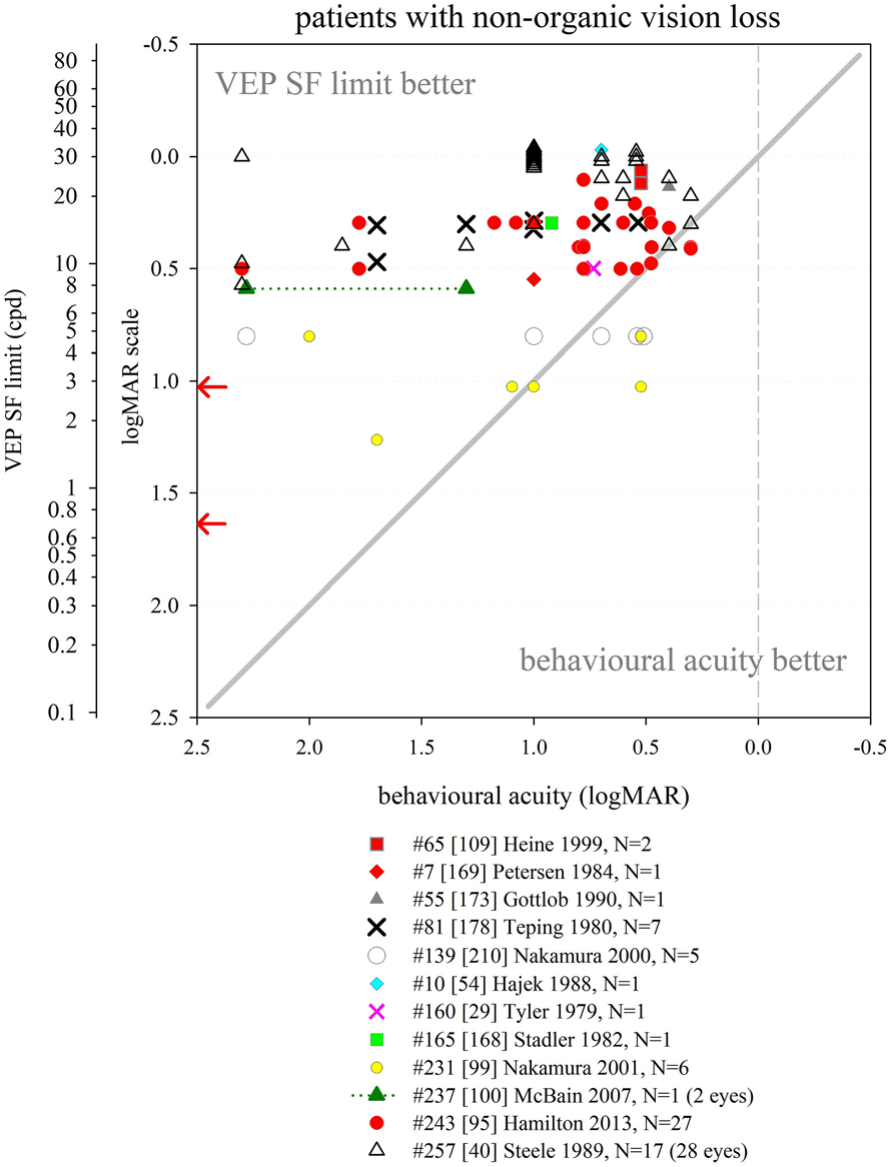
VEP SF limit versus behavioural acuity in patients with non-organic visual acuity loss. Solid grey line: equality. Dashed grey line: indicative normal behavioural acuity limit (0.0 logMAR). Red arrows at the SF axis indicate the two ISCEV standard checkwidths, 60′ and 15′ (0.71 and 2.8 cpd)

Thirteen studies were identified where patients with, or suspected of having, NOVL had both behavioural acuity and VEP SF limit documented, 12 with extractable data (Fig. 18). All VEP data are presented in terms of SF limit, either extrapolated or finest SF, rather than in terms of a “corresponding acuity”. Even without applying such adjustments, VEP SF limits were almost universally better than behavioural acuity. Two studies with moderately sized groups showed normal VEP SF limits for all patients, despite behavioural acuities as poor as 2.3 logMAR [40, 95]. For 27 children (5–15 years) with NOVL, VEP SF limits were 0.54 (range 0.11–2.79) log units better than behavioural acuity. One further child had visual perceptual difficulties, an optotype acuity of 5/12 (0.380 logMAR) and VEP SF limit of 15 cpd (within local reference limits), illustrating the inability of VEP SF limits to reflect higher visual processing difficulties [95]. In 28 eyes of 17 patients (7–68 years), VEP SF limits were significantly better than Snellen acuities and the authors noted that VEPs was the method able to “definitively and objectively diagnose functional visual loss” [40]. Similar findings were noted in smaller groups or in individual patients, whether children or adults [29, 54, 109, 168, 169, 173, 178, 209]. The great majority of a large group (*N* = 100) of patients with suspected NOVL were found to have an extant VEP to the finest SF used (5.5′ checkwidth, 7.7 cpd) regardless of contrast. This VEP was evident even at low (20%) contrast, a finding associated with behavioural acuities of − 0.079 to 0.000 logMAR in 10 healthy adults tested with the same protocol [100]—illustrative data from both eyes of one patient are shown in Fig. 18.

Two studies found generally poor VEP SF limits but nonetheless concluded that VEP SF limits were in keeping with better behavioural acuities than reported by patients [99, 210]. For five children (7–14 years) suspected to have NOVL with interpretable VEPs [210] and for six adults suspected of malingering [99] all had VEP SF limits (finest SF technique) at a 9′, low-contrast checkwidth (4.7 cpd, 0.804 logMAR; 15%), which the authors note corresponds to a behavioural acuity of “nearly” 0.000 logMAR.

While all VEP SF limit assessments should be conducted as one part of a full ophthalmic and electrophysiological assessment, this may especially be the case for suspected NOVL. As can be seen from Figs. 12, 13 and 14, normal VEP SF limits might be obtained in the presence of retinal, macular or optic nerve pathologies. Unless all possible organic causes for visual loss have been ruled out by ophthalmic and electrophysiological investigations, interpreting a normal VEP SF limit as confirmation of NOVL could miss sight-threatening pathology.

#### Other conditions

VEP SF limits have been described in relatively small numbers of patients with a variety of other ophthalmic conditions not already described.

Strabismus: Fifteen infants (10–50 weeks), otherwise typically developing, with untreated esotropia and alternating fixation had monocular and binocular VEP SF limits which fell 0.30 and 0.23 log units, respectively, below the averages for age-matched control infants, but they did not have significant interocular VEP SF limit differences [211]. Four 2-year olds with strabismus and alternating fixation but without amblyopia were found to have VEP SF limits (0.68–0.13 logMAR) which quite closely matched behavioural acuities measured several months later. The authors concluded that VEP SF limits accurately predicted future recognition acuity [186].

Glaucoma: In seven patients with open-angle glaucoma, VEP SF limits and behavioural acuities showed very good correlation: the difference was within 0.30 log units for 6/7 patients. One example patient, a 40-year-old man with optic nerve head cupping, had behavioural acuity of 0.000 logMAR and a VEP SF limit of 16.1 cpd (0.27 logMAR). [172]. In 12 patients with glaucoma, those with intact fields (*N* = 5, 8 eyes) showed close correlation between VEP SF limit and behavioural acuity: however, if visual field defects were evident (*N* = 4), no VEPs were evident even to the coarsest SF (96′ grating, i.e. 0.31 cpd, 1.98 logMAR) despite behavioural acuities between 0.18 and 0.48 logMAR [40].

Albinism: In 13 children aged 0.1–10 years with albinism and foveal hypoplasia (11 also with nystagmus), VEP SF limits using horizontal, sinusoidal gratings ranged from 0.176 to 1.176 logMAR, generally poorer than typical for age (cf Fig. 6) [212]. Two patients with albinism had better VEP SF limits than behavioural acuities by around 0.1 and 0.3 log units [187]. An 8-year-old boy with albinism including nystagmus had behavioural acuity of 1.000 logMAR and VEP SF limits about 0.1 log units better [173], and a 3-year-old patient with albinism had a VEP SF limit 0.08 log units better than behavioural acuity [55]. Limited data on the presence or absence of foveal hypoplasia and/or nystagmus, and grouping of data across ages when VEP–behavioural differences are likely to change markedly, preclude drawing conclusions about VEP SF limits in albinism.

Down syndrome: Researchers established a VEP SF limit in 16/28 (57%) of young children with Down syndrome and in 91% of age-matched control children. VEP SF limits and behavioural acuities were 0.2–0.3 log units poorer in children with Down syndrome, not entirely attributable to attention or accommodation effects, suggesting a primary sensory deficit [158].

Autistic spectrum disorder (ASD): Sixteen children (5–17 years) with ASD, no learning disability and corrected-to-normal visual acuity had the same VEP SF limits as an age-matched control group (24.6 vs. 25.8 cpd; 0.086 vs. 0.066 logMAR). The ssVEP second harmonic in children with ASD was smaller across mid-range SFs (5–17 cpd), especially at the right occipital electrode, which the authors suggested reflects compromise of a highly specific neural substrate early in the visual pathway [72].

Vigabatrin-treated infantile spasms: VEP SF limits were investigated in a group (*N* = 42) of children with infantile spasms using, or who had used, vigabatrin. Presence of vigabatrin-related retinal toxicity was presumed if two or more consecutive flicker ERGs had reduced age-corrected amplitude relative to baseline and relative to previous recording by more than the inter-visit variability. VEP SF limits were poorer in those with presumed retinal toxicity (*N* = 10) than in those without (*N* = 32): 0.42 versus 0.27 logMAR. Expressed relative to mean VEP SF limits of age-matched controls, children with presumed retinal toxicity had poorer VEP SF limits by 0.144 log units, while those without had better limits by 0.032 log units [213].

## Discussion

It is clear from the large body of literature systematically reviewed here that the VEP SF limit has been applied widely but has yet to be extensively accepted as an objective acuity estimator. One reason for this may be the lack of a standardised protocol and hence, in some cases, widely disparate findings from different laboratories. Another reason is the difficulty of interpretation: for example, what does a VEP SF limit of 10 cpd mean? The findings presented here clearly indicate that it does not mean the same thing in an adult with cataract as in a child with optic nerve hypoplasia or a baby with poor visual behaviour.

The International Society for Clinical Electrophysiology of Vision (ISCEV) writes and updates standards [66, 214–217], guidelines [218, 219] and extended protocols [220–224] with the aim of reducing inter-laboratory test variability, one aspect of quality improvement which reduces inherent uncertainty and enhances patient safety. The ISCEV standards, guidelines and extended protocols address quality of the test process, provide some guidance on appropriate clinical use, and address some aspects of interpretation and communication of test results. An extended protocol (“specifications for specialized procedures that are sufficiently well established and that have broad acceptance by experts in the field”) for estimating acuity with VEPs is in press [225].

The challenge associated with VEP SF limit interpretation has its origins in psychophysics, where traditionally a threshold stimulus with a fundamental SF of 30 cpd has been associated with a visual acuity of 0.000 logMAR, i.e. 1.0 (decimal), 6/6 or 20/20 (Snellen). This relationship often fails to hold for VEP SF limits, as illustrated in Figs. 5 and 7. We conclude that it is misleading and inaccurate to arithmetically convert the fundamental SF of the limiting VEP stimulus into the units and terminology of perceptual (behavioural) acuity as this would imply a direct relationship which is accurate in only limited cases. For the same reason, we have reservations about the expression “VEP acuity” because of the risk that non-expert clinical users will expect the same capability of patients as they would from letter acuity. Retaining units of cpd and establishing reference values and critical limits in those units for clinical reports might circumvent this issue. An alternative approach is to quantify the empirical relationship between an optimised VEP SF limit technique and psychophysical (or behavioural) acuity results: certainly in adult patients tested with robust methodology, there is good evidence for the validity of this approach. However, this empirical calibration is highly dependent on age, VEP technique and—especially for paediatrics—acuity test. Furthermore, the relationship between VEP SF limit and behavioural acuity obtained from artificially blurred, healthy adults (Fig. 8) does not necessarily accurately emulate findings from patients with visual acuity loss (e.g. Fig. 9), although Fig. 9 reflects many diverse methodologies, not all necessarily robust. Precision is also an issue: where this has been assessed, the limits of agreement between VEP SF limit and behavioural acuity, even when calibrated empirically, are typically ± 0.3 log units (± 1 octave) and can be much wider. This precision should be established via reference values and quoted and interpreted in clinical reports.

Assuming adequate technical reporting of a VEP SF limit, including its age-specific accuracy and precision, clinical interpretation presents a further challenge. For reasons outlined in the introduction, a VEP SF limit has not assessed the same aspect of vision as a clinical acuity test. Good examples of wording which avoids the misleading impression that the VEP SF limit equates to behavioural acuity include: “the presence of a response to a pattern stimulus implies that the visual system contains elements capable of resolving the stimulus” [117]. In conjunction with an empirical calibration, “with [X%] likelihood the visual resolution measured from early cortical visual processing corresponds to an acuity of better than [value in, e.g. logMAR]” [12]. The latter example also notes the possibility of an organic disorder affecting higher visual areas or NOVL.

The data collated here from studies of patients with visual acuity loss suggest that VEP SF limits are a good proxy for behavioural acuity in several conditions, where “good” is defined as accurate over a range of acuities, with reasonable precision. Such conditions include patients with media opacities, refractive errors and primarily retinal dysfunction.

Where the primary site of dysfunction is the macula, the optic nerve or any cerebral structures, VEP SF limits have poorer accuracy and precision when compared to behavioural measures. In macular disease, the foveal dominance of the VEP is evident in mostly poor VEP SF limits, while the wide scatter presumably reflects general reduction of VEP amplitudes, giving rise to large errors in extrapolated VEP SF limits (Fig. 13). Optic nerve disease similarly presents VEP SF limits poorer than behavioural acuity with wide scatter, again perhaps due to generally low VEP amplitudes impairing limits and increasing error (Fig. 14).

A large volume of data from amblyopic patients confirm that VEP SF limits are relatively insensitive to reduced optotype acuity in amblyopia—many patients with behavioural acuities as poor as 1.0 logMAR continue to show VEP SF limits similar to those of patients with near-normal acuity (Fig. 15). Because the VEP can define a task-free SF threshold which is relatively robust to higher neural noise and to fixation problems [55], it has been used as a predictive marker for outcomes following therapy: however, VEP SF limits prior to amblyopia therapy, being typically better than behavioural acuity, may simply roughly coincide with later, improved levels of optotype acuity. Diagnostically, VEP SF limits are poorly sensitive to amblyopic acuity loss; therefore, a poor VEP SF limit may indicate incorrect refraction, or subtle macular or optic nerve pathology. Interestingly, the pattern of VEP SF limit versus behavioural acuity seen in amblyopic patients is similar, albeit less extreme, to that seen in children with neurological causes for their vision loss, especially CVI (Figs. 16, 17), i.e. VEP SF limits are generally better than behavioural acuity, with the difference more apparent at poor acuities. This not only reinforces the neurological nature of amblyopia, but also raises the issue of which test should be regarded as the gold standard. For some children with neurological impairment, the additional burden of recognition and motor responses required by behavioural tests might mean the VEP SF limit represents their visual threshold more accurately than an acuity card test based on preferential-looking. In contrast, for children with amblyopia, VEP SF limits may be an inaccurate reflection of their visual capabilities because VEP gratings or checkerboards are relatively robust to the “phase-scrambling” effect.

Non-organic vision loss (NOVL) has received much attention from clinicians working with VEP SF limits, and the data compiled here support its use in this area (Fig. 18). The importance of age-specific reference data with pre-specified accuracy and precision is particularly relevant since some workers state the VEP SF limit to be normal even when the finest SF assessed is relatively large [99, 210], and when similar VEP SF limits have been reported in the presence of retinal, macular or optic nerve pathologies (cf Figs. 12, 13, 14). Unless all possible organic causes for visual loss have been ruled out by ophthalmic, neurological, imaging and electrophysiological investigations, interpreting a within-reference-limits VEP SF limit as confirmation of NOVL could miss sight-threatening pathology. Furthermore, a normal VEP SF limit in combination with poor acuity may indicate dysfunction of higher visual processing areas rather than NOVL; such patients may benefit from event-related potential threshold measures [226]. In a very small number of extreme cases, patients with no behavioural vision at all can present with extant and even normal pattern VEPs [202, 227].

Perhaps the highest utility of VEP SF limits, other than in NOVL, lies in paediatric testing, whether in pre-verbal children or children with motor or learning impairments which prevent reliable measurement of behavioural acuity. As with all paediatric tests, the diagnostic power of a VEP SF limit depends heavily on adequate, age-stratified, reference data from typically developing infants and children [228]. The data presented here indicate that typical limits increase from 1 to 20 cpd over the first year of life, then increase more slowly to reach adult levels between 2 and 10 years of age (Fig. 6). Success rates are variable, but are better for shorter test protocols. The most marked difference between paediatric and adult VEP SF limits is seen when they are compared with behavioural acuities: as reported by many authors, VEP SF limits are much better than behavioural acuity in the youngest, typically developing infants, but the reverse is found from around 3–5 years, with behavioural acuity being somewhat better than VEP SF limits, as seen in adults. This makes it critical that any empirical calibration of VEP SF limits is established for all ages, and that adult calibrations are not applied to infants or to children younger than around 3–5 years. In a typical adult, a reasonable degree of association between the VEP SF limit and the perceptual threshold is to be expected; in most cases, the resolution threshold for the optical system and visual pathways is similar and perception and attention are not limiting factors. The size of the VEP generated in the occipital cortex reduces approaching the SF limit, representing diminished cortical activation, and extrapolation to zero or to noise indicates absence of a cortical signal and thus a threshold for the entire visual system. The story is different during development: although infant visual sensitivity is limited by optical, photoreceptor, foveal and neural immaturity [229], these do not fully account for the fact that VEP SF the SF limits are far better than behavioural acuity in the first months of life. Two possible reasons have been suggested [230]. Firstly, signals encoding high SFs might be available at the visual cortex to be tapped by the VEP, but then be altered or lost in higher processing centres and therefore unavailable to be tapped by behavioural tests. Secondly, some small signals may be detectable as a VEP following repetitive stimulation and averaging but in behavioural tests, an infant’s response is required trial-by-trial with no opportunity for summing stimuli. There is no accepted gold standard technique for measuring infant visual acuity, and both behavioural and electrophysiological measures are flawed. Preferential-looking tests are neurally demanding and require motor responses which may depress thresholds; however, it is not justifiable to assume an infant can “see” a stimulus which evokes a VEP but which does not elicit a behavioural response. On the other hand, if the VEP SF limit is within the reference range for age and protocol, it is reasonable to infer that the visual pathway from optics to cortex is intact. This holds true regardless of the patient's age (although it may be less true for certain pathologies). For this reason, VEP SF limits, and indeed VEPs, are uniquely valuable for assessing the integrity of the early visual pathway.

Regardless of how a patient presents, a VEP SF limit cannot stand alone, but must be ordered and interpreted in the light of the neonatal, ophthalmological, neurological and neuroradiological, imaging and electrophysiological context of each patient, and with a full understanding of the implications of the VEP protocol including age-specific reference data. The importance of using VEP SF limits as only part of a fuller assessment cannot be overstressed. VEP SF limits cannot be interpreted without full clinical assessment and history; assessment should often include standard clinical electrophysiological testing (full field, flash and/or pattern ERGs, VEPs, etc.), ocular and neural imaging techniques and other diagnostic testing. Its clinical use should be reserved exclusively for patients who cannot or will not cooperate or satisfactorily complete behavioural acuity tests or whose cooperation is suspect: behavioural tests have real-life meaning and are almost always the gold standard. An exception might be if the clinician understands why the VEP SF limit and behavioural acuity might differ, but seeks their complementary information.

Visual electrophysiology laboratories without specific thresholding procedures but which record ISCEV standard VEPs to checkwidths of 60′ and 15′ (0.71 and 2.8 cpd), and often to additional pattern sizes, may attribute an acuity according to the presence or absence of these transient VEPs. For example, the statement “good visual acuity means good sized response to smallest checks (6.25′)” was included in a recent consensus statement, in the context of whether a baby or very developmentally delayed child could see [231]. A checkwidth of 6.25′ has SF_f_ of 6.8 cpd: an extant VEP (transient or steady state) to this SF would certainly be in keeping with “good visual acuity” for a baby, but would be poorer-than-typical for any patient aged over 1 year. It is normal for subjects aged over a few weeks to have a VEP to the ISCEV standard 60′ (0.71 cpd) checkwidth, and normal for subjects over 6 months to have a VEP to the ISCEV standard 15′(2.8 cpd) (cf Fig. 6) [232, 233]. Depending on the diagnostic question, one strategy might be to record both a rapid, objective, ssVEP SF limit and transient VEP(s) to pattern sizes informed by the VEP SF limit, or vice versa. This strategy is short enough to have a reasonable chance of maintaining patient engagement while also capturing the rich diagnostic information in the parameters and waveshapes of transient VEPs. For other patients, prioritising transient VEPs, including monocular testing, may be the better strategy to make best use of limited cooperation and attention, and using the VEP SF limit as a valuable adjunct or separate assessment.

This systematic review has several shortcomings. We have undertaken little quality or bias assessment of included studies, principally because no standard outcome measure or intervention was being reviewed. The included studies had widely divergent purposes: many did not address either of the questions of this review as their principal aim. We have indicated as far as possible factors such as number of subjects or patients included. A further shortcoming is the great variety of stimulus, recording and analysis techniques and combinations employed in the included studies, so the effect of altering one parameter such as age is always confounded in other studies by multiple other differences.

The future for VEP SF limits in the clinical setting is promising. Hugely increased, inexpensive computational power has enabled multiple improvements to even the most robust and accurate techniques described here. For example, significance testing and threshold extrapolation could be performed in real time with stopping rules to minimise test duration and to give feedback control for subsequent stimulus presentation [92, 234]. Increased dwell time in low SNR conditions could enhance accuracy and precision of VEP SF limits. SsVEPs are extensively used in the field of brain–computer interfaces (BCI), where accuracy and speed of detection—information transfer rate in BCI language—are essential to reduce user frustration. Principal component analysis, independent component analysis and canonical correlation analysis are widely used for signal detection in BCI [235, 236], but not so far in the field of VEP SF limits. Combining eye tracking with ssVEPs is providing more robust user navigation and takes advantage of the reducing costs and current rapid evolution of both EEG and eye tracking data acquisition components [237]. Eye tracking combined with VEP SF thresholding could automatically restrict analysis to only those EEG epochs captured when the patient is looking at the stimulus, or could use gaze-deviation to trigger an attention-grabbing change of stimulus or sound to help re-establish fixation. Machine learning has already been demonstrated to improve VEP SF limit estimation [238], an approach which could help bypass choices of threshold definition and calibration, and could even use individual sweeps to obviate the need for feature extraction. Finally, event-related potentials [226] and fixation- or saccade-related potentials [239] could bridge the gap between cortical and cognitive SF limits.

## Abbreviations

VEP: Visual evoked potential
ssVEP: Steady-state VEP
SF: Spatial frequency
SF_f_: Fundamental SF
deg: Degree
MAR: Minimum angle of resolution
logMAR: Logarithm (base 10) of MAR
cpd: Cycles per degree, also written as cy/deg or cy/°
DFT: Discrete Fourier transform
SNR: Signal-to-noise ratio
rps: Reversals per second
EEG: Electroencephalogram
LoA: Limits of agreement
ETDRS: Early treatment diabetic retinopathy study (acuity test)
SEM: Standard error of the mean
SD: Standard deviation
ASD: Autistic spectrum disorder
CP: Cerebral palsy
PVL: Periventricular leukomalacia
TAC: Teller acuity cards
NOVL: Non-organic vision loss

## Footnotes

1 Note this section discusses linear versus exponential *sampling* of SF. The separate, but related, issue of linear versus logarithmic SF axis scaling is discussed.
